# Deciphering the sexual diploid members of the *Boechera suffrutescens* complex (Brassicaceae, Boechereae)

**DOI:** 10.3897/phytokeys.98.24296

**Published:** 2018-05-02

**Authors:** David P. Morin, Patrick J. Alexander, James B. Beck, Michael D. Windham, C. Donovan Bailey

**Affiliations:** 1 Biology Department, New Mexico State University, P.O. Box 30001 – MSC 3AF, Las Cruces, New Mexico 88003, USA; 2 Department of Biological Sciences, Wichita State University, 1845 Fairmount, Wichita, Kansas 67260, USA; 3 Botanical Research Institute of Texas, 1700 University Drive, Fort Worth, TX 76107, USA; 4 Department of Biology, Duke University, 139 Biological Sciences (Box 90338), Durham, North Carolina 27708, USA

**Keywords:** *Boechera duriuscula*, *Boechera botulifructa*, Apomixis, Taxonomy, Sexual Diploid

## Abstract

*Boechera* is a model genus that is of particular interest for understanding apomixis due to the presence of numerous apomictic diploid lineages that are tightly correlated with hybridisation events. *Boechera* includes many narrowly distributed endemics and apomictic hybrid lineages that obscure morphological boundaries amongst taxa. In this study, we focus on the *Boechera suffrutescens* complex, a phylogenetically well-supported but taxonomically complex north-western United States clade whose diploid species currently include the widespread *B. suffrutescens* and two narrowly distributed serpentine endemics, *B. constancei* and *B. rollei*. Using a 15-locus microsatellite dataset, we infer ploidy and sexual vs. apomictic reproduction for all individuals and then assess species limits for all sexual diploid samples. Our results support the recognition of *B. rollei* and *B. constancei* as distinct species and reveal three divergent sexual diploid lineages within *B. suffrutescens* sensu lato. The latter three lineages exhibit geographic, genetic and morphological coherence and consequently warrant recognition at the species rank. These include *Boechera suffrutescens* s.s., which is restricted to Idaho and eastern Oregon, *Boechera botulifructa*, a newly described species distributed along the Cascade Mountain Province from Lassen County, California north to Deschutes County, Oregon and the heretofore dismissed species *Boechera duriuscula* (basionym ≡ *Arabis duriuscula*), which occurs along the Sierra Nevada Province from Plumas County southwards to Fresno County, California. Our data also reveal substructure in *B. constancei* that is likely attributable to the highly fragmented distribution of its serpentine habitat. This refined taxonomic framework for the *B. suffrutescens* complex enhances *Boechera* as a model system, adds to our knowledge of speciation in edaphically extreme environments and provides information on ongoing conservation efforts for these taxa.

## Introduction

The genus *Boechera* Á.Löve & D.Löve was first recognised in 1976, but it was not widely accepted as distinct from *Arabis* L. prior to 2003 (Al-Shehbaz 2003). This largely North American lineage represents a complex assemblage of ±83 sexual diploid (S2X) taxa that have given rise to hundreds of apomictic hybrids, a situation that has confounded morphological classification since the first species were described in the 1820s (Li et al. 2017). The causes of this complexity include recent divergence and ongoing hybridisation, limited morphological disparity, edaphic shifts and the presence of both apomictic diploid (A2X) and triploid (A3X) hybrid lineages that are common and persistent across the distribution (Beck et al. 2011; Windham and Al-Shehbaz 2006; Windham and Al-Shehbaz 2007a; 2007b). *Boechera* is nearly unique amongst flowering plants in possessing numerous diploid apomictic lineages (Bicknell and Koltunow 2004; Koltunow and Grossniklaus 2003). Understandably, the confluence of these characteristics has attracted considerable attention and *Boechera* has become a focal point for studies of biogeography, speciation, adaptation, apomixis and ecological genomics.

Despite widespread interest in *Boechera* as a model system (e.g. Rushworth et al. 2011) ongoing research has been hindered by limited understanding of species-level diversity, biogeography and phylogeny. Only recently, through a combination of molecular phylogenetic and population genetic studies, has genuine progress been made towards a coherent *Boechera* classification. This has involved a modified “diploids first” approach (Brown et al. 2002), acknowledging that it is nearly impossible to identify and study apomictic hybrids without an in-depth understanding of the sexual diploid species that gave rise to them. This approach has proven highly effective in *Boechera*, documenting cryptic biodiversity and bringing new clarity to both the *B. fendleri* and *B. lignifera* species complexes (Alexander et al. 2015). Here, we apply this method to another poorly known group, the *B. suffrutescens* complex.

**Figure 1.**
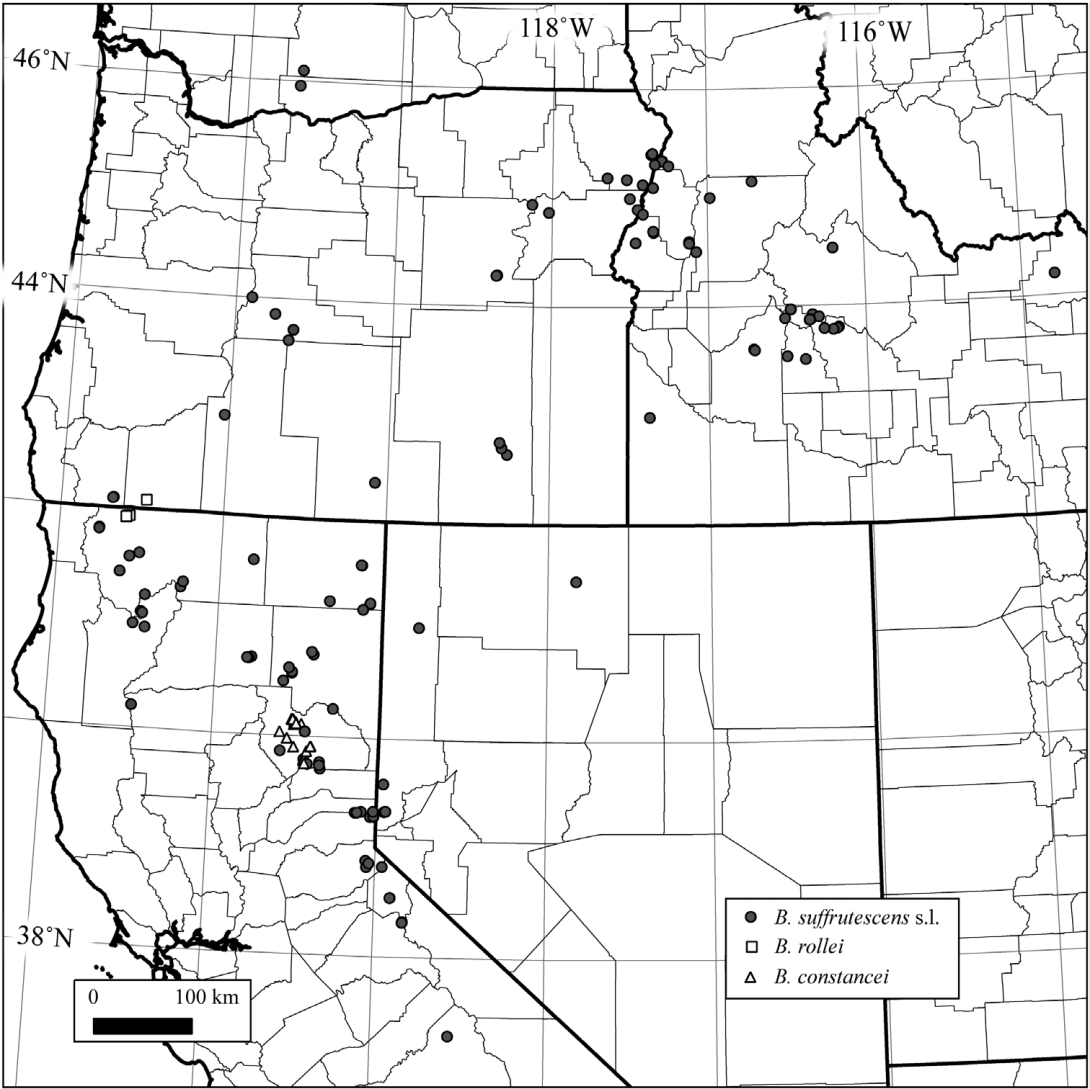
Geographic distribution of 498 initial individuals. Only 307 diploids were retained for diploid-level molecular analyses. All maps were created in QGIS (Quantum GIS 1137 Development Team 2013).

The ability to distinguish amongst different ploidy levels and reproductive modes in *Boechera* rests on several well-documented correlations derived from chromosomal, microsatellite heterozygosity and pollen data (Alexander et al. 2015; Beck et al. 2011; Li et al. 2017). Initially, pollen morphology was used as the primary indicator (Al-Shehbaz and Windham 2010; Windham and Al-Shehbaz 2006; Windham and Al-Shehbaz 2007a; 2007b). S2X lineages produce pollen in tetrads through normal meiosis; the individual grains are mostly uniform, narrowly ellipsoid, 13–16 μm wide, with three symmetrical colpi (Suppl. material 1: fig. 1A). A3X lineages produce any functional pollen in dyads by means of apomeiosis; these grains are more irregular, ovoid-spheroid, 22–30 μm wide, with more than three asymmetric colpi (Suppl. material 1: fig. 1B) (Windham and Al-Shehbaz 2006) . A2X lineages usually produce predominantly malformed pollen (resulting from irregular meiotic events) mixed with functional meiotic and/or apomeiotic pollen (Suppl. material 1: fig. 1C) (Beck et al. 2011). More recently, an extensive 15-locus microsatellite database, encompassing nearly all known sexual diploid taxa and over 4400 accessions (Li et al. 2017), has made it possible to determine both ploidy level and reproductive mode through microsatellite analysis on a simple DNA sample. This dataset has confirmed previous reports of a bimodal distribution of heterozygosity across the genus (Alexander et al. 2015; Beck et al. 2011). Comparative meiotic studies of over 134 individuals representing 84 lineages of *Boechera* reveal that the left peak of this bimodal distribution (heterozygosity <0.5) consists almost entirely of S2X individuals while the right peak includes mostly apomicts. Amongst the apomicts, A3X lineages can then be distinguished from A2X lineages by the presence of three alleles at one or more of the 15 microsatellite loci (Alexander et al. 2015; Beck et al. 2011).

Our improved ability to sort *Boechera* specimens into natural groups, combined with cluster analysis of microsatellite data and phylogenetic analysis of DNA sequence data, have greatly improved our understanding of several S2X species complexes (Alexander et al. 2015; Windham et al. 2015). Nevertheless, there are many groups that require additional study to characterise extant sexual diploid diversity. One such group is the *B. suffrutescens* complex. This complex currently includes three S2X species (*B. constancei* (Rollins) Al-Shehbaz, *B. rollei* (Rollins) Al-Shehbaz and *B. suffrutescens* (S. Wats.) Dorn) that formed a maximally supported clade in genus-wide molecular phylogenetic analyses (Alexander et al. 2013). Two A3X species (*B. horizontalis* (Greene) Windham & Al-Shehbaz and *B. rigidissima* (Rollins) Al-Shehbaz) are believed to be hybrids between members of the *B. suffrutescens* complex and more distantly related species of *Boechera* (Al-Shehbaz and Windham 2010).

The group takes its name from *Arabis suffrutescens* S. Wats., which has been broadly defined to include populations from the Sierra Nevada, Trinity Alps, Cascades and isolated mountain peaks across the northern Great Basin, southern Columbia Plateau and Rocky Mountains of central Idaho. This highly variable taxon includes both S2X and A3X populations (Al-Shehbaz and Windham 2010), which occur in close proximity near the type locality along the Snake River Gorge in eastern Oregon. Eighteen years after Watson named *A. suffrutescens*, Greene described a segregate species, *Arabis duriuscula* Greene. The taxon was typified based on collections from Donner Lake, California, which Rollins (1941) subsequently treated as a taller and less suffrutescent phenotype of *A. suffrutescens*. *Arabis dianthifolia* Greene, described from the vicinity of Crater Lake (Greene 1910), has also been viewed as synonymous with *A. suffrutescens* (Al-Shehbaz and Windham 2010).

Two other taxa were segregated from *Arabis suffrutescens* by Rollins (1993b) and subsequently transferred to *Boechera* by Windham and Al-Shehbaz (2006). *Boechera rollei* is the most narrowly distributed taxon in the group, known only from the Trinity Mountains in Siskiyou County California (Fig. 1) and an isolated population along upper Beaver Creek, Jackson County, Oregon. The relative showiness of its flowers indicates that it is likely an outcrossing S2X lineage (Schmidt and Bancroft 2011). The other commonly accepted segregate is *B. constancei*, a narrow endemic apparently confined to Plumas and Sierra Counties, California. This taxon is known to be diploid based on a published chromosome count from the type locality (Rollins and Rüdenberg 1971) and it exhibits protogyny with distinctly elongated styles, which is suggestive of outcrossing (Schmidt and Bancroft 2011). In addition to being of conservation concern, both *B. constancei* and *B. rollei* appear to be restricted to serpentine soils, a model substrate for studying the links between edaphically extreme environments and divergent plant speciation (Kruckeberg 1951; 1984; 2002).

Although *B. constancei* and *B. rollei* are generally separable from the wide-ranging *B. suffrutescens*, there are some collections that appear to be morphologically intermediate (Rolle, pers. comm.). There also are unresolved questions regarding the placement of *Arabis duriuscula* and *A. dianthifolia* in synonymy under *Boechera suffrutescens*, as well as the relationship between S2X and A3X populations of the latter (Al-Shehbaz and Windham 2010). The purpose of this study is to identify and characterise the S2X lineages (taxa) within the *B. suffrutescens* complex to provide a framework for future investigations into the origins of related A2X and A3X lineages. Along with traditional macro-morphological and pollen analyses, we apply the set of 15 microsatellite loci, previously employed by Beck et al. (2011) and Alexander et al. (2015), to both herbarium specimens and extensive recent field collections. Pollen and microsatellite data are used to infer the ploidy and reproductive mode of each accession. The S2X individuals singled out by this process are used in a series of population genetic analyses to identify genetically coherent lineages worthy of species-level recognition.

## Materials and methods

### Sampling

Samples for the project were obtained from individuals representing the morphology and known geographic range of the complex (Fig. 1), including 150 newly added collections and 348 previously collected herbarium samples. Holotype specimens of *Arabis suffrutescens*, A. suffrutescens var. perstylosa, *A. rollei*, *A. constancei* and an isotype of *A. duriuscula* were included. The holotype of *A. dianthifolia* was observed online through the Smithsonian plant database (https://collections.nmnh.si.edu).

### Correlation between pollen morphology and reproductive mode

In concert with other data, pollen morphology was used to assign or confirm the assignment of individual plants to S2X, A2X or A3X categories. Pollen samples of adequate quality were obtained from 45 individuals and were analysed and categorised following Beck et al. (2011). Pollen was mounted in glycerol and immediately observed, characterised and photographed on an Olympus CH-2 objective microscope at various magnifications.

### DNA extraction

Genomic DNA was extracted using a modified version of the protocol outlined in Alexander et al. (2015). The deviation included: dried leaf samples being homogenised without buffer, eluting the pulverised material in grinding buffer plus 12 µl (>600 mAU/ml) of Proteinase K and an incubation with agitation at 65 °C for 12–15 hours prior to moving on to the next step.

### Amplification of microsatellite loci

Fifteen previously published microsatellite loci (ICE3, ICE14 (Clauss et al. 2002), BF3, BF9, BF11, BF15, BF18, BF19, BF20, Bdru266 (Song et al. 2006), a1, a3, b6, c8 and e9 (Dobes et al. 2004) were amplified via five multiplex polymerase chain reactions (PCR) following Beck et al. (2011). Forward primers were 6-FAM or HEX labelled. Amplicons were sized with the 500 LIZ standard (Applied Biosystems Corp., Carlsbad, CA) on an Applied Biosystems 3730 xl at the University of Chicago Comprehensive Cancer Center DNA Sequencing and Genotyping Facility. Allele sizes were determined using GeneMarker 1.91 (SoftGenetics, State College, PA). Locus a3 was excluded from downstream analyses due to potential unresolved paralogy consistent with the findings of Beck et al. (2011) and Alexander et al. (2015).

### Identification of S2X individuals and populations

All samples with data for at least 8 of the 15 microsatellite loci were retained for analysis. The ploidy level of each sample was then estimated using the criteria outlined by Beck et al. (2011). In short, if an individual exhibited no more than two alleles per locus, it was inferred to be diploid; if three alleles were present at one or more loci, it was inferred to be triploid. Following Alexander et al. (2015), the mode of reproduction amongst diploids (e.g. S2X vs. A2X) was then inferred via an average number of alleles per non-null locus (ANA/NNL) approach. The S2X category was initially set to a mean ANA/NNL ≤1.5 (Alexander et al. 2015) and later reduced to ≤1.35 or less following downstream population genetic analyses that identified hybrid “*B. constancei*” with values above 1.35 (discussed below).

### Analyses of population structure within and amongst S2X taxa

Following Alexander et al. (2015), we employed a hierarchical approach to investigate patterns of microsatellite variation and population differentiation amongst putative species-level lineages. STRUCTURE employs a parametric Bayesian approach to investigate the most likely number of differentiated (*K*) population systems (Falush et al. 2003; Pritchard et al. 2000). Exploratory STRUCTURE analyses were performed with the admixture model using default settings with 50,000 burn-in and 500,000 post-burn in generations with five iterations at each value of *K* from 1–11. Final STRUCTURE analyses included 100,000 burn-in and 1,000,000 post-burn in generations with 10 iterations for each value of *K* from 1–11. The most likely value of *K* for each analysis was identified using the ∆*K* method of Evanno et al. (2005) as implemented in STRUCTURE *Harvester* (Earl and vonHoldt 2012). Null alleles at seven loci (B11, C8, BF15, Brdu266, e9, BF3 and BF19), clearly corresponding to either prior taxonomic assignment and/or geographic structure, were coded in STRUCTURE using RECESSIVE ALLELES = 1 (Falush et al. 2007).

AWclust utilises a nonparametric approach to infer population structure based on allele sharing distance (Gao and Starmer 2008). Critically, this approach does not incorporate a model of within-group Hardy-Weinberg equilibrium, an assumption that is likely unrealistic considering the interspecific, biogeographic and temporal (inclusion of historical specimens) scope of our sample set. Multidimensional scaling plots (MDS) were generated in AWclust to visualise relative coherence and distinctness of clusters based on allele sharing distance. AWclust estimates the optimal number of clusters (*K*) via the gap statistic (Tibshirani et al. 2001), whereby individuals are assigned to clusters at the optimal *K* through the implementation of Ward’s minimum variance hierarchical clustering. Gap statistics were calculated for a given data set with 100 null simulations for *K* values 1–11. The aforementioned null alleles were also treated as characters in AWclust.

### Morphological assessment a posteriori

Individuals inferred as representing S2X species-level lineages through the aforementioned analyses were subsequently studied in detail to identify diagnostic morphological characteristics for the taxonomic treatment.

## Results

### Correlation between pollen morphology and reproductive mode

Forty-five accessions harboured pollen of sufficient quality and quantity for morphotyping. Of these, 26 individuals exhibited ovoid-spheroid, multicolpate pollen consistent with apomictic reproduction, 14 exhibited narrowly elliptic, tricolpate pollen consistent with sexual reproduction and five exhibited presumably non-viable pollen with a spheroid, ecolpate morphology. There was 92% agreement between mode of reproduction inferred via pollen morphology and that inferred by the maximum number of alleles per locus (see below). This high correlation is consistent with prior studies by Beck et al. (2011), who reported a 96% correlation in a sample of 330 specimens.

### Identification of S2X individuals and populations

The maximum number of alleles per locus criterion (Alexander et al. 2015; Beck et al. 2011) identified 191 triploid (which were excluded from further analysis (see Appendix 1)) and 307 diploid (S2X and A2X) individuals (see Table 1, “Additional specimens examined” and Appendix 1). For ease of data presentation in Table 1 and this text, populations are represented by abbreviations that include a locality prefix (CD = Canyon Dam, CP = Cascade Province, GB = Great Basin, OVR = Onion Valley Reservoir, PLSI = Plumas and Sierra Counties, SNP = Sierra Nevada Province, TL = type locality region,) and a species suffix (co = *constancei*, ro = *rollei* and su = *suffrutescens* s.l.). If the specific identifier is preceded by an ‘x’ (e.g. OVR-xco), the group is a putative hybrid lineage assigned to the A2X category. Ploidy assignment for a small number of individuals was inconsistent with prior inferences for their taxon. In particular, 9 of 96 *B. constancei* individuals were inferred to be triploids despite prior diploid inference from a smaller sample of individuals (Rollins and Rüdenberg 1971).

### Subsequent analyses focused on differentiating A2X and S2X individuals

The 1.5 ANA/NNL criterion (Alexander et al. 2015; Beck et al. 2011) identified 238 putative S2X individuals. Preliminary STRUCTURE runs were then employed to fine-tune the ANA/NNL cutoff. These preliminary studies identified nine putative population systems (excluding “singletons”) with 16–59 individuals per group (S2X and A2X in Table 1). A small subset of three individuals from Falcon Valley, Washington did not cluster with other population systems. “Falcon Valley” is an anomalous place name used by W.N. Suksdorf and we are unable to determine from where these specimens were collected. Given the geographic uncertainty and poor sampling of this lineage, plants from “Falcon Valley” were excluded from further analysis. A group of individuals (OVR-xco) from Onion Valley Reservoir that is morphologically assignable to *B. constancei* showed genetic admixture. These individuals, inferred to represent a previously undetected A2X hybrid lineage, exhibited a mean ANA/NNL of 1.36. In light of this, the S2X mean cutoff was reset to <1.35 to provide a more conservative circumscription of the S2X category. After applying these filters, we were left with 235 inferred S2X individuals to be included in the final analyses.

**Table 1.** Summary of sexual diploid (S2X), apomictic diploid (A2X), and apomictic polyploid (A3X and A4X) assignments and clusters inferred from preliminary analyses. Polyploids, A2X clusters, and singletons were excluded from the final S2X analyses (see text).

Clusters											
	CD-co	CP-su	TL-co	PLSI-co	OVR-xco	SNP-su	TL-su	TL-ro	GB-xsu	Single-tons	Poly-ploids
# individuals	18	38	18	35	16	59	31	36	48	8	191
Inferred Reproductive mode	S2X	S2X	S2X	S2X	A2X	S2X	S2X	S2X	A2X	A2X, S2X	A3X, A4X
Mean ANA/NNL with range	1.295 (1.0–1.5)	1.004 (1.000–1.083)	1.044 (1.000–1.091)	1.150 (1.000–1.300)	1.362 (1.077–1.500)	1.087 (1.000–1.385)	1.142 (1.000–1.462)	1.180 (1.000–1.462)	1.547 (1.385–1.692)	1.471 (1.182–1.667)	2.006 (1.167–2.750)
# Individuals with analyzed pollen or meiotic counts	7	1	–	4	–	2	3	8	2	–	30

### Analyses of population structure within and amongst S2X taxa

**Figure 2.**
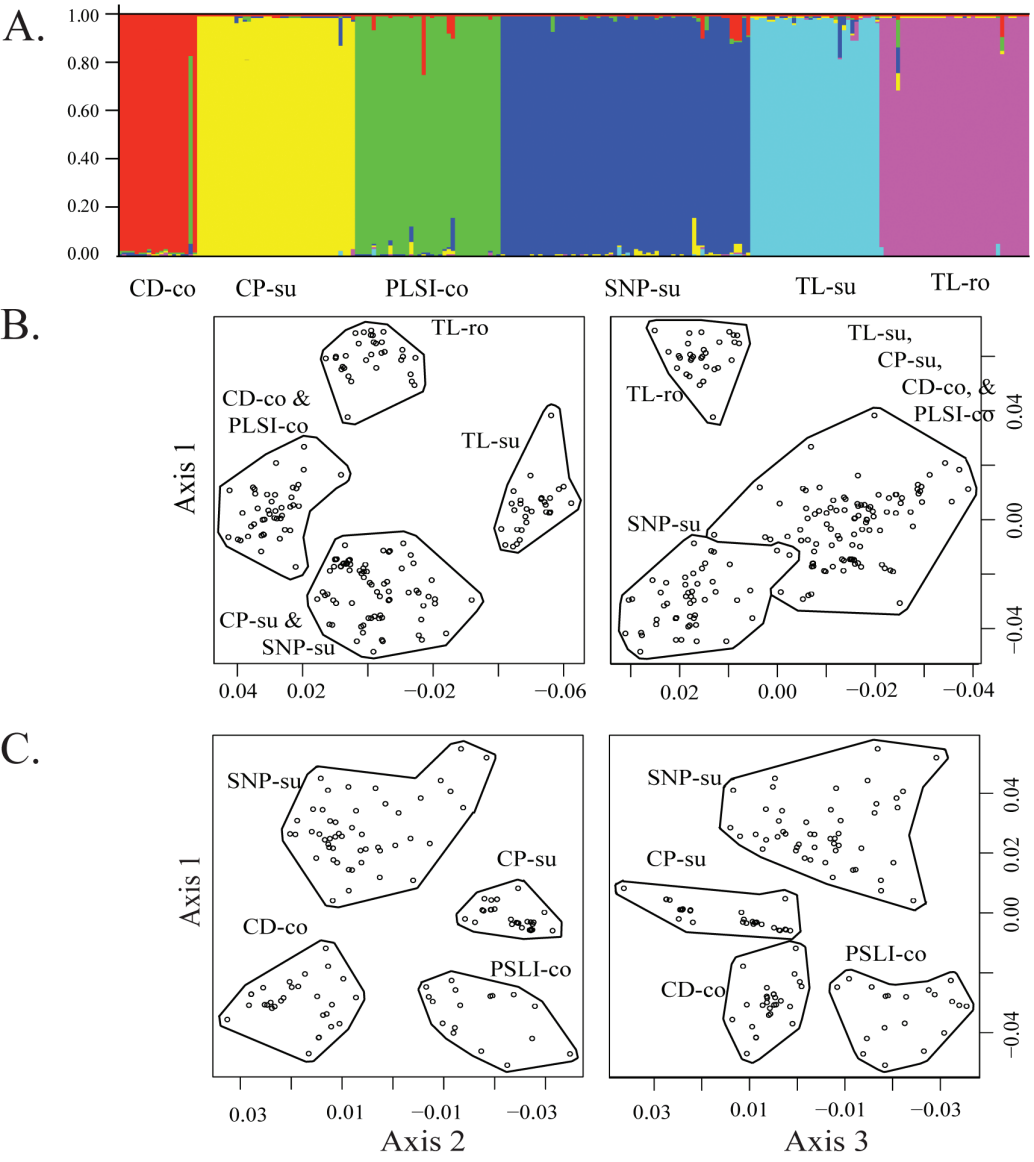
Results from Analysis 2 (which excluded TL-co). **A** STRUCTURE bar plot with K = 6 **B** MDS microsatellite plot of first three axes at K = 6 as found with the gap statistic **C** MDS plot with TL-su and TL-ro, the most divergent clusters, excluded to demonstrate coherence of the remaining four clusters (gap statistic K = 4).

**Figure 3.**
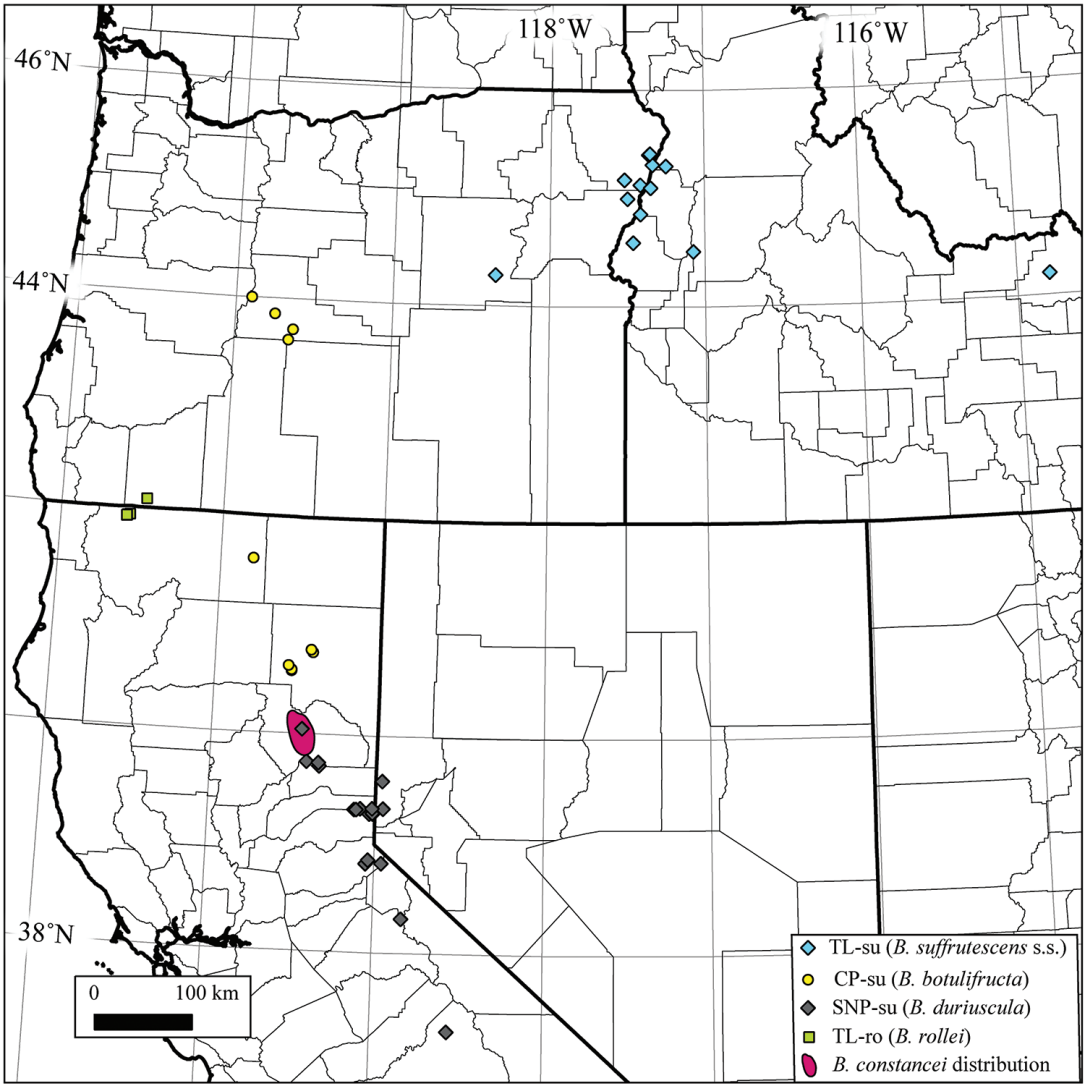
Geographic distribution of *B. suffrutescens* (CP-su, SNP-su, and TL-su) and *B. rollei* (TL-ro) lineages inferred from Analysis 2. A map of Plumas Co., California with distributions of the B. constancei clusters is presented in Fig. 4.

**Figure 4.**
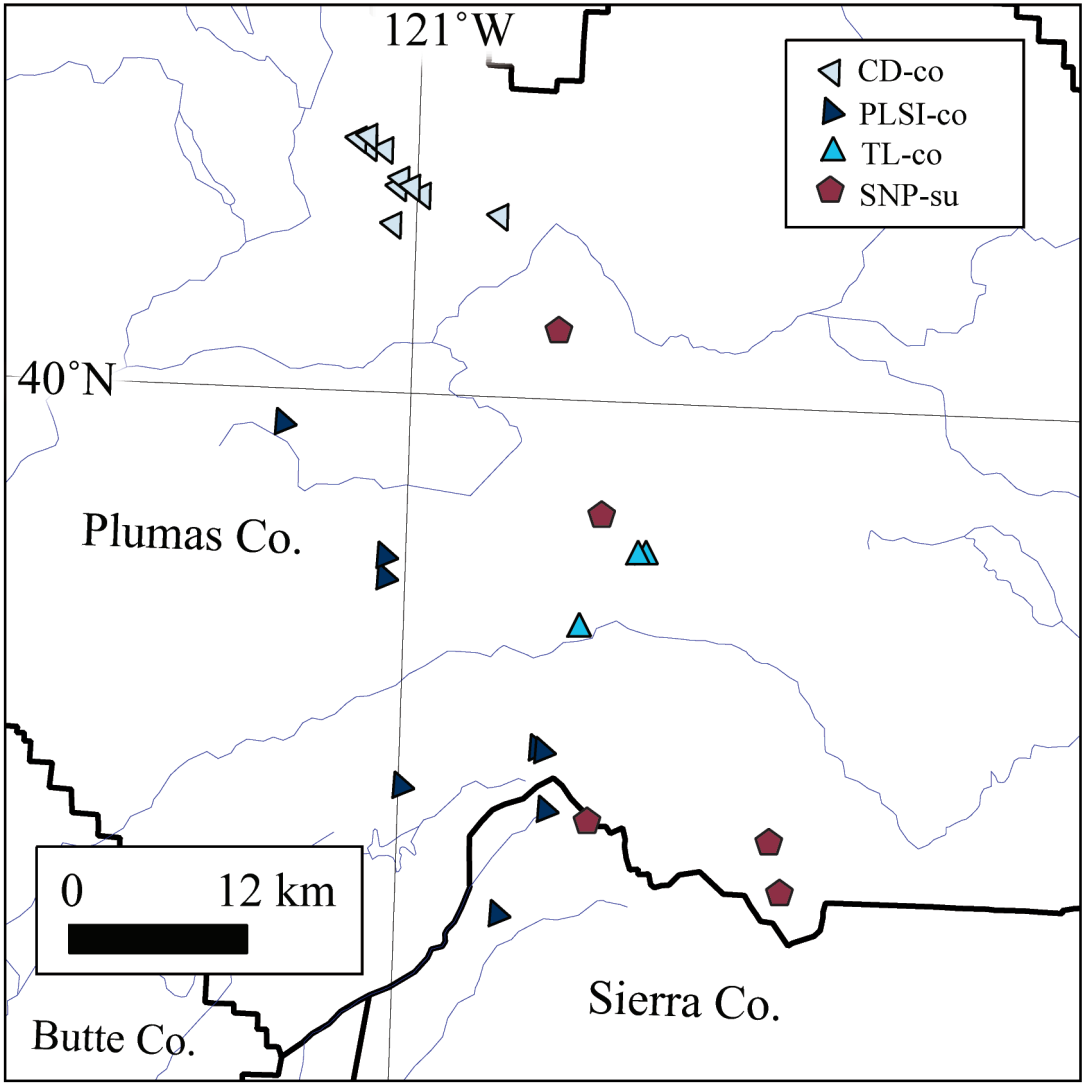
Distribution of diploid *B. constancei* s.l. and SNP-su clusters in Plumas County, California. CD-co lies to the north of the east branch of the north fork of the Feather River. The type locality cluster (TL-co) exhibited unstable placement in both genetic clustering analyses.

Our diploid only (see above) and preliminary S2X only analyses revealed conflict and instability in the optimal *K* inferred by STRUCTURE as well as between AWclust and STRUCTURE. Analysis 1, including all 235 S2X individuals, yielded two equally optimal *K* (3 and 8) in STRUCTURE and two equally optimal *K* values (4 and 8) in AWclust. The instability observed within and between these analyses was the result of conflicting assignments for individuals of *B. constancei* from the type locality (TL-co). TL-co individuals either formed a unique cluster (Suppl. material 2: fig. 2A, *K* = 6 and 8) or occasionally grouped with CP-su individuals (Suppl. material 2: fig. 2A, *K* = 5). These findings were consistent with potential introgression involving TL-co and CP-su. Given the instability associated with TL-co, we performed a second round of analyses (Analysis 2) without TL-co, which yielded an unambiguous *K* = 6 from both STRUCTURE and AWclust. This array specifies *B. rollei* as a single cluster, but supports two distinct clusters (CD-co and PLSI-co) within *B. constancei* and three distinct clusters (CP-su, SNP-su and TL-su) within *B. suffrutescens* s.l. (Fig. 2). Each of these clusters also occupies a discrete geographic range (Figs 3, 4) with possible introgressant populations (TL-co) located in close proximity to the most similar putative parent (*B. constancei*) but nearly 100 km south of the documented range of the other (CP-su).

### Morphological Assessment a Posteriori

Morphological comparisons of the clusters identified by STRUCTURE and AWclust revealed a variety of features useful for distinguishing these putative taxa. Character state differences in petal length, style length and the presence or absence of auricles on the cauline leaves have been used to separate *B. rollei* and *B. constancei* from *B. suffrutescens* s.l. Each of these features is consistent with differentiation amongst these lineages. Additionally, we have identified a series of morphological features that support the recognition of the three clusters belonging to *B. suffrutescens* s.l. (CP-su, SNP-su, and TL-su). These include basal leaf pubescence, shape of the fruit apex and the length-to-width ratio of mature basal leaves (see “Taxonomic Account” section below).

## Discussion

### Assignment of ploidy and characterisation of S2X

The stepwise process employed to first parse diploids from polyploids and then S2X from A2X, identified 238 S2X individuals out of a total sample of 498 *B. suffrutescens* complex samples. The fact that more than half of the individuals were A3X or A2X clearly illustrates that the *B. suffrutescens* complex harbours the hybridisation, apomixis and polyploidy trifecta that has severely hindered species-level taxonomy in the genus as a whole (Alexander et al. 2015; Windham and Al-Shehbaz 2007a; 2007b). The analytical approach taken here supports the recognition of five sexual diploid taxa within the *B. suffrutescens* complex. These lineages include the current circumscriptions of *B. constancei* and *B. rollei* and a recircumscription of *B. suffrutescens* that recognises three distinct taxa. Each of these five taxa is discussed below.

### *Boechera rollei*

All 36 individuals of *Boechera rollei* were clearly defined as S2X by allele numbers (Table 1) and as a distinct group by cluster analyses (Fig. 2). The taxon is very rare, with just three known populations restricted to serpentine soils in Siskiyou County, California and Jackson County, Oregon. Its distribution overlaps with the broadly distributed *B. suffrutescens* s.l. (Fig. 1), but the two have not been observed growing together. *Boechera rollei* is separable morphologically from all other S2X members of the complex by its unusually large (8–11 mm long) cream-coloured (vs. lavender) petals and non-geniculate fruiting pedicels. It is further separable from *B. constancei* by its auriculate cauline leaves and shorter styles (≤1.5 mm). Genetics, ecology, geography and morphology all support recognition at the species level, a conclusion that is consistent with prior taxonomic treatments (Al-Shehbaz and Windham 2010; Rollins 1993a) and meets the criteria proposed by the genetic species concept (Baker and Bradley 2006; Bateson 1909), the phylogenetic species concept (Nixon and Wheeler 1990) and the genotypic cluster concept (Mallet 1995) for species level recognition.

### *Boechera suffrutescens* sensu lato

*Boechera suffrutescens* s.l. is by far the most widespread and morphologically heterogeneous taxon in the complex. With regard to the S2X lineages, STRUCTURE and AWclust analyses subdivided individuals identified as S2X *B. suffrutescens* s.l. into three geographically distinct clusters (CP-su, SNP-su and TL-su) with little or no evidence of admixture (Fig. 2 and Table 2). The population system (TL-su) that includes the type locality for *B. suffrutescens* is S2X based on ANA/NNL results (mean 1.142) and pollen morphology (Table 1). It is separable form other S2X lineages based on several lines of evidence. It is genetically distinctive, forming a cohesive cluster in both *STRUCURE* and AWclust analyses (Fig. 2) and it exhibits unique fixed alleles at both the e9 and BF9 microsatellite loci. Its geographic range, extending ca. 100 km north-south along the Idaho-Oregon border with outlying populations in Fremont County, Idaho and Grant Counties, Oregon (Fig. 3), is separate from those of the other S2X taxa. This lineage is morphologically distinctive as well, characterised by having narrower basal leaves (length/width ratio usually ≥8:1) and sparser pubescence relative to the CP-su and SNP-su lineages (see “Taxonomic Treatment”). The combination of these features support recognition of this cluster as *B. suffrutescens* s.s.

The two remaining S2X lineages currently included within *B. suffrutescens* s.l. are distributed from the southern Sierra Nevada north into the central Cascades (Fig. 3). Both exhibit mean ANA/NNL values and pollen morphologies consistent with S2X assignment (Table 1). Like the TL-su cluster, members of the SNP-su group formed a cohesive cluster in both STRUCTURE and AWclust analyses (Fig. 2). Our sampling, comprising 59 individuals representing 23 populations of SNP-su, is distributed along the Sierra Nevada from Fresno to Plumas Counties, California (Fig. 3). At the northern end of its range, SNP-su overlaps with the distribution of *B. constancei* and hybridisation between these taxa may have given rise to the presumed A2X lineage OVR-xco (see discussion below). The SNP-su cluster is separable from sympatric populations of *B. constancei* by having shorter styles (≤1.5 mm) and auriculate cauline leaves and is distinguished morphologically from the other *suffrutescens* s.l. S2X taxa by fruit and pubescence characters. This cluster included an isotype of *Arabis duriuscula* Greene, a taxon that has been treated as a synonym of *B. suffrutescens* (e.g. Al-Shehbaz 2003; Al-Shehbaz and Windham 2010; Rollins 1993a; Windham and Al-Shehbaz). The findings presented here support the recognition of a distinct taxon requiring the recognition of *Arabis duriuscula* at the species level in *Boechera* (see “Taxonomic Treatment”).

**Table 2.** Summary S2X only analyses one and two. ‘X’ indicates inclusion in the analysis using both AWClust and STRUCTURE.

Clusters											
Formal Analysis	CD-co	CP-su	TL-co	PLSI-co	SNP-su	TL-su	TL-ro	# of Individuals	ΔK	Gap Statistic K	Result
1	X	X	X	X	X	X	X	235	3.8	5.7	Unstable
2	X	X	–	X	X	X	X	219	6	6	K = 6

The second group previously assigned to *B. suffrutescens* s.l., the CP-su cluster, is represented by 38 individuals. Like the other two *suffrutescens* s.l. clusters, CP-su is genetically distinct in both STRUCTURE and AWclust analyses (Fig. 2). Current sampling suggests that it occupies a discrete geographic range in the Cascade Mountain Province extending from Lassen and Siskiyou Counties, California to Deschutes County, Oregon (Fig. 3). In addition to being genetically and geographically distinct, members of the CP-su cluster are separable from the other S2x taxa previously assigned to *B. suffrutescens* s.l. based on the distinctive, ovoid-shaped fruit apices. None of the species-level names, previously formalised in *Boechera* or *Arabis*, appear to be applicable to this taxon and we therefore propose a new name: *B. botulifructa* (see “Taxonomic Treatment”).

### *Boechera constancei*

Previous studies of the obligate serpentine endemic *B. constancei* considered it S2X based on chromosome counts (Rollins and Rüdenberg 1971) and pollen morphology (Al-Shehbaz and Windham 2010). Amongst our sampling of 87 individuals representing 28 unique geographic sites, 71 samples from 26 localities were indeed assigned to the S2X category based on ANA/NNL ratios (Table 1). The 16 samples from Onion Valley Reservoir (OVR-xco) were inferred to be A2X. Even with OVR-xco removed, population genetic analyses including all S2X *B. constancei* revealed considerable instability. STRUCTURE and AWclust analyses recovered as many as three clusters, CD-co, PLSI-co and TL-co (Fig. 4). The apparent instability was associated with an affinity between TL-co and CP-su, potentially indicative of hybridisation or introgression between these S2X groups. The species is well separated morphologically from the other S2X members of the *B. suffrutescens* complex by its unusually long styles (≥1.5 mm) and consistently non-auriculate cauline leaves.

The isolated island-like biogeography (Ellstrand and Elam 1993; Young et al. 1996) of *B. constancei* on serpentine soils could explain the patterns of geographically defined genetic sub-structure observed in this taxon (Suppl. material 2: fig. 2B). Future work is especially needed to investigate the complex substructure observed in *B. constancei* and to determine whether segregate taxa worthy of recognition are contained within the species.

### Evaluation of A2X lineages in the *Boechera suffrutescens* complex

Two major A2X clusters were evident in the complex. The aforementioned OVR-xco cluster consisted of 16 individuals collected from serpentine in the vicinity of Pilot Peak and Onion Valley Reservoir in Plumas County, California. The STRUCTURE allele assignment profiles for these individuals exhibited apparent admixture between SNP-su and *B. constancei* and they resolved in intermediate positions between these putative parents in AWclust plots (data not shown). No pollen data were available from which to infer mode of reproduction. Further research will be required to confirm this assertion and investigate the origin of this cluster.

The second major A2X cluster (GB-xsu) is broadly distributed across the mountain ranges of the Great Basin in California, Oregon and Idaho. It is morphologically assignable to *B. suffrutescens* s.l. Both the mean ANA/NNL of GB-xsu and pollen morphology from two individuals indicate that this is an A2X lineage (Table 1). Some additional preliminary analyses suggest it may have arisen through hybridisation between SNP-su and CP-su (Windham, unpubl. data).

The focus of this study was on circumscribing the sexual diploid taxa of the *B. suffrutescens* complex and our combined data support the recognition of at least five S2X species. Although not discussed in detail here, at least two A2X lineages and an even greater diversity of A3X hybrid lineages were also evident. More than half of the individuals included in this study ultimately were assigned to asexual groups and preliminary analyses imply that some of these lineages incorporate one or more genomes from outside of the *B. suffrutescens* complex (Morin, unpubl. data). This is consistent with prior observations that hybridisation and a transition to apomixis may be linked (e.g. Beck et al. 2011) and that recognising the impact of these phenomena is a critical part of deciphering *Boechera* diversity and evolution.

## Taxonomic treatment

Members of the *B. suffrutescens* complex are distinguished from congeneric taxa by having relatively wide (2.5–6 mm) pendent fruits containing a single row of broadly winged (0.3–1.5 mm) seeds per locule. Previous molecular analyses (Alexander et al. 2013) have established that sexual diploid accessions of *B. constancei*, *B. rollei* and *B. suffrutescens* s.l. (represented by the SNP-su lineage) form a well-supported clade. In addition to the five S2X taxa characterised below, we encountered many A2X and A3X individuals. Some of these exhibit morphological characters that clearly set them apart from S2X taxa, suggesting that they are products of hybridisation with other species groups. More problematic were the apomictic hybrids that have arisen within the *B. suffrutescens* group, blurring the already subtle distinctions amongst the S2X members of the complex. It should be noted that the taxonomic treatment provided below applies only to S2X individuals and that pollen morphology and/or allelic diversity are the only reliable means for distinguishing closely related sexual and apomictic lineages in *Boechera* as a whole (Beck et al. 2011; Windham and Al-Shehbaz 2006).

### Key to sexual diploid taxa of the *B. suffrutescens* complex

Given the frequency of hybridisation in *Boechera*, pollen morphology should be characterised prior to proceeding with this key. An inference of sexual diploidy can be made for individuals that produce mostly well-formed, narrowly ellipsoid symmetrically tricolpate pollen (Suppl. material 1: fig. 1). In terms of the macromorphological characters used in the key, there is considerable variation within species and some inevitable morphological overlap between species. Multiple plants should be examined if possible. *Caute procedere*.

**Table:** 

1	Petals mostly more than 6 mm long, cream white, but occasionally with rose-coloured apices (*B. rollei*); mature fruiting pedicels curved-descending or reflexed but never distinctly geniculate proximally; plants only known from serpentine (ultramafic) substrates in the Klamath Mountains or on the west slopes of the Sierra Nevada near Lake Delahunty	**2**
–	Petals mostly less than 6 mm long, usually lavender-purple but occasionally cream with rose-coloured apices; many mature fruiting pedicels distinctly geniculate proximally, more or less straight distally; plants found mostly on non-serpentinic (felsic) substrates across a wide range from the southern Sierra Nevada north through the central Cascade Province and east to central Idaho	**3**
2	Upper cauline leaves with distinct auricles 0.5–2.5 mm long; styles 0.5–1.5 mm long; petals 8–11 mm long; fresh herbage without a distinct bluish cast. Klamath Mountains	***Boechera rollei***
–	Upper cauline leaves without auricles; style 1.5–5.5 mm long; petals 6–8 mm long; fresh herbage usually with a distinct bluish cast. Plumas and Sierra Counties in the vicinity of Lake Delahunty	***Boechera constancei***
3	Basal leaves on most plants glabrous or glabrate with few 1–2(3) rayed trichomes on the leaf margins and apices; length-to-width ratio of mature basal leaves usually ≥8:1; plants distributed from Grant County, Oregon east to central Idaho	***Boechera suffrutescens***
–	Basal leaf surfaces pubescent and the leaves ciliate, with 2–4(–5) rayed trichomes; length-to-width ratio of mature basal leaves 4:1–9:1; plants of the Sierra Nevada and Cascade Provinces	**4**
4	Mature fruit apex abruptly tapered into an ovoid tip with an apical angle (measured from the style base to a point 5 mm proximal to it) mostly greater than or equal to 30°; plants distributed in the Cascade Province from Lassen County, California to Deschutes County, Oregon	***Boechera botulifructa***
–	Mature fruit apex more gradually tapered, with an apical angle (measured from the style base to a point 5 mm proximal to it) less than 25°; plants found in the Sierra Nevada from Fresno County, California north to Plumas County, California and near Lake Tahoe in Washoe County, Nevada	***Boechera duriuscula***

#### Boechera botulifructa

Taxon classificationPlantaeBrassicalesBrassicaceae

D.P. Morin sp. nov.

urn:lsid:ipni.org:names:60476298-2

Figures 3 , 5

##### Type.

**U.S.A. California.** Lassen County: 1.75 mi SSE of Coulthurst Flat on E road cut berm of Champs Flat Road. 1.35 air mi SSW of Cleghorn Reservoir, 26 Jun 2012, *C.D. Bailey & D.P. Morin 24* (holotype: NMC!; isotypes: DUKE!, MO!).

##### Diagnosis.

As a member of the *B. suffrutescens* complex, *B. botulifructa* can be distinguished from most other species of *Boechera* by pendent relatively wide siliques (2–6 mm). Within the complex, the species is one of just five that produces narrowly ellipsoid symmetrically tricolpate pollen (Suppl. material 1: fig. 1A) indicative of diploid sexual reproduction. *Boechera botulifructa* is distinguishable from four other sexual diploid *B. suffrutescens* complex species by the presence of small petals (4–6 mm long), abruptly tapered silique distal apices and a geographic distribution along the Cascade Province in California and Oregon.

##### Description.

Plants long-lived perennials, with woody caudices raised above ground level 1–5 cm, lacking crowded, persistent leaf bases; herbage without an obvious bluish cast. Fertile stems 1(–3) per caudex branch, each arising from a basal rosette, lower parts pubescent to densely pubescent with short-stalked, 2–3(4) rayed trichomes 0.1–0.3 mm. Leaves: at stem bases oblanceolate, 1.7–5.8 mm wide, entire, ciliate with 2–3(–4) rayed trichomes to 0.07–0.40 mm; cauline leaves (4–)6–12, occasionally concealing stem proximally, the uppermost glabrous, with auricles (0)0.3–1.4 mm. Inflorescences mostly unbranched, 6–12 flowered; mature fruiting pedicels 9–17 mm, reflexed, distinctly geniculate proximally, but straight distally, glabrous. Flowers pendent at anthesis; sepals glabrous; petals 4.5–6.0 long × 2.0–2.5 mm wide, pale lavender or whitish with rose apices; anthers with mostly well formed, narrowly ellipsoid, symmetrically tricolpate pollen; ovules 20–30 per fruit. Fruits 3–7(–10) cm long × 2.0–2.5 mm wide, pendent, straight to somewhat curved, with undulate edges; apical angle of fruit valve 30–38° (measured from base of style to 5 mm proximate); style persistent 0.2–1.2 mm long. Silique apex mostly rounded apically. Seeds uniseriate, 2.5–5.5 × 1.8–3.5 mm; wing continuous, 0.8–1.5 mm wide.

##### Distribution, habitat and phenology.

As currently known, the species occupies three distinct regions in the Cascade Province: western Deschutes County, Oregon, near Medicine Lake, Siskiyou County, California and the area west of Eagle Lake in Lassen County, California. It favours rocky slopes and gravelly felsic soils in association with *Artemisia tridentata*, *Purshia tridentata*, *Pinus jeffreyi*, *Pinus contorta* and *Juniperus* at elevations of 1300–2100 m; flowering from May to July.

##### Comments.

Morphologically, *B. botulifructa* is most similar to *B. duriuscula* and these two taxa are parapatric along the southern distribution of *B. botulifructa*. The species is distinguished from close relatives primarily by the abrupt tapering of the apex of the fruit, resulting in a sausage-like profile to which the specific epithet refers. Like most other members of the *B. suffrutescens* complex, it has a suffrutescent habit and wide (>3 mm), reflexed, often secund, fruits. Molecular data suggest that the specimens from the southernmost population in Lassen County, California, have diverged from the northern populations and may have a history of gene flow with *B. constancei* from the vicinity of its type locality. The latter *B. botulifructa* individuals, from Lassen County, also have reduced cauline leaf auricles relative to other non-serpentinicolous members of the complex.

Though the species spans a large geographic range, we have only identified nine populations systems thus far, suggesting a need for future investigation of conservation status. Within the populations we visited, individuals were sparsely dispersed across broad areas. The species occurs on public lands with noted impacts from grazing activity and potential impacts from logging of local native forests.

The holotype for *Arabis dianthifolia* Greene was collected at Crater Lake, Oregon, but our access to this type was limited to high resolution images. Although Crater Lake lies within the overall range of *B. botulifructa*, the specimen observed lacked the diagnostic fruit apex character. Furthermore, preliminary microsatellite analyses of specimens collected near the type locality indicate that *A. dianthifolia* is probably A2X (Windham, unpubl. data).

##### Specimens examined.

**California.** Lassen County: Pine Creek near Bogard Ranger Station, 23 Jun 1960, *S.K. Harris 21448* A, B (GH); USFS 22N02. 1.25 road mi N of highway 44, 26 Jun 2012, *D.P. Morin 22* A - I (NMC); 1.75 mi SSE of Coulthurst Flat on E road cut of Champs Flat Road. 1.35 air mi SSW of Cleghorn Reservoir, 26 Jun 2012, *D.P. Morin 24* A, B, C (NMC), D, E, F, (DUKE), G, H, I (MO); Coulthurst Flat area (T34N, R10E, S27, SW), 29 Jun 1983, *G.D. Schoolcraft 1038* (NY). Siskiyou County: Medicine Lake, 28 Jul 1921, *A. Eastwood 10885* A, B, C (GH). **Oregon.** Deschutes County: Along Elk Lake, 13 Jun 1925, *C.H. Peck 14337* (WILLU); Take unnamed dirt road E 0.4 mi from Jones Well Rd. 7 air mi SSW of Paulina Lake, 26 Jun 2012, *D.P. Morin 17* A, B, C, D - J (NMC); Deschutes NF, Ann’s Butte, ca. 3.5 mi W of Sunriver on Rd. 40, 26 Jun 1992, *D.W. Taylor 12889* A, B (NMC); Paulina Lake, 29 Jul 1894, *J.B. Leiberg 584* A, B, (OSC).

**Figure 5.**
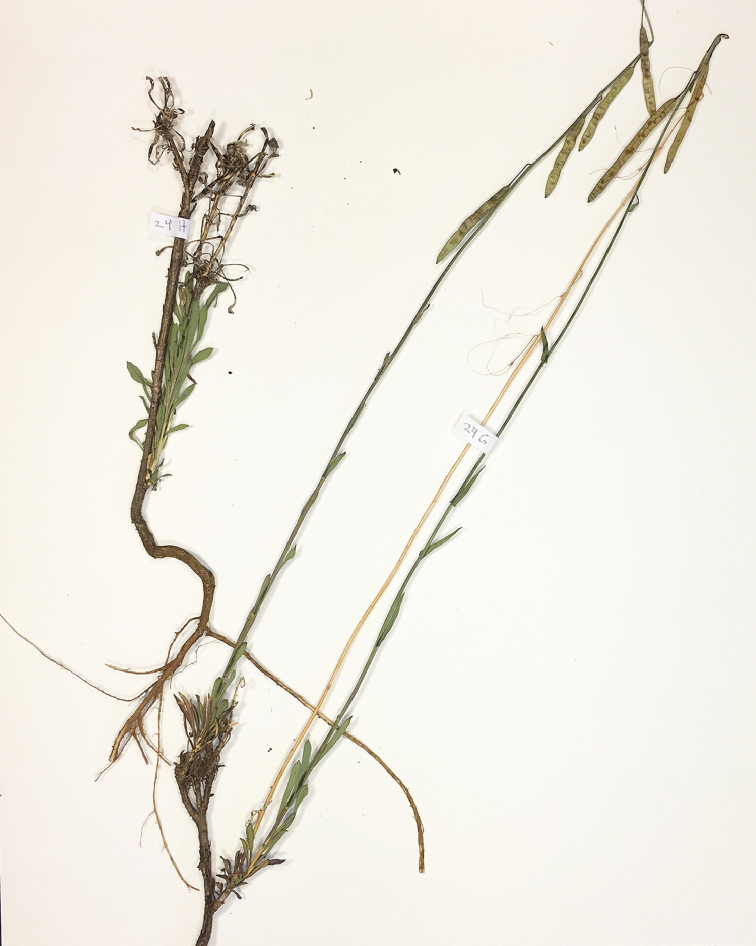
*Boechera botulifructa*, Morin 24 (MO).

#### Boechera constancei

Taxon classificationPlantaeBrassicalesBrassicaceae

(Rollins) Al-Shehbaz, Novon 13: 384. 2003.

Figures 3 , 4 , 6

≡ Arabis constancei Rollins, Contr. Gray Herb. 201:5. 1971. **Type: U.S.A**. **California**. Plumas County: 7.6 mi SE of Quincy (at Spring Garden Overpass), on road to Blairsden, 11 Jul 1969, *L. Constance* and *T. Chuang 3875* (holotype: GH!; isotype: UC!). GH holotype image – http://kiki.huh.harvard.edu/databases/specimen_search.php?mode=details&id=49339
A. =A. suffrutescens S. Watson var. perstylosa Rollins, Rhodora 43: 471. 1941. **Type: U.S.A**. **California**. Plumas County: Above the Middle Fork of the Feather River 7.3 mi SE of Quincy, 9 Jun 1938, *L. Constance 2309* (holotype: GH!; isotypes: DS, NY, UC!, WS, WTU). GH holotype image – http://kiki.huh.harvard.edu/databases/specimen_search.php?mode=details&id=122692

##### Description.

Plants long-lived perennials, with woody caudices raised above ground level 1–5 cm, lacking crowded, persistent leaf bases; herbage often with a distinct bluish cast. Fertile stems 1(–3) per caudex branch, each arising from a basal rosette, 1.2–3.0 dm, glabrous or glabrate proximally with few 1–2 rayed trichomes. Leaves at stem bases narrowly oblanceolate, 1.5–4.0 mm wide, entire, ciliate with simple and stalked 2–3(4) rayed trichomes 0.3–0.8 mm, basal leaf surfaces glabrous with ciliate margins to pubescent; cauline leaves 6–12, glabrous, lacking auricles, usually not concealing the stem proximally. Inflorescences unbranched, 5–15 flowered; mature fruiting pedicels 4–12 mm, strongly recurved or reflexed proximally but not distinctly geniculate proximally, glabrous. Flowers divaricate-ascending at anthesis; sepals glabrous; petals 6–8 mm long × 1.5–2 mm wide, creamy white, glabrous; anthers with mostly well formed, narrowly ellipsoid, symmetrically tricolpate pollen; ovules 18–28 per fruit. Fruiting pedicels glabrous, 5–15 mm, recurved but not distinctly geniculate proximally. Fruits 3.6–7.5 cm long×3.0–3.5 mm wide, pendent or reflexed, usually secund, straight or slightly curved, with undulate margins, glabrous; apical angle of fruit valve 16–25° (measured from base of style to 5 mm proximate); style persistent 1.5–5.5 mm. Seeds uniseriate, 3–4 × 2.5–3 mm; wing continuous, 0.5–1.0 mm wide.

##### Distribution, habitat and phenology.

*Boechera constancei* is only known from the western slope of the Sierra Nevada in the vicinity of Lake Delahunty in Sierra County and adjacent southern Plumas County, California. It appears to be confined to a variety of serpentinic substrates in association with *Pinus jeffreyi* and other “serpentine barren” vegetation types at elevations from 1200–1900 m; flowering from Apr–Jun.

##### Comments.

*Boechera constancei* was originally treated as a variety of *Arabis suffrutescens* s.l., but it is distinguished from members of that group by its non-auriculate cauline leaves, longer (6.0–8.0 vs. 4.5–6.0 mm) petals that are creamy white and longer (1.5–5.0 vs. 0.4–1.2 mm) style. Although restricted to serpentine substrates, it generally shows greater local abundance and higher population densities within its narrow geographic range than *B. duriuscula*, *B. botulifructa* and *B. suffrutescens* s.s.

##### Specimens examined. California.

Plumas County: Central Sierra Nevada, Plumas National Forest, N side of F.R. 24N20 above East Branch Rock Creek, 1.0 road miles from the junction with 24N28, 1.1 miles NE of Deanes Valley Campground, 4.3 miles WSW of central Quincy, 4 Aug 2009, *P.J. Alexander 997* A (DUKE), B, E, F (NMC); Above the Middle Fork of the Feather River, 7.3 mi SE Quincy, 9 Jun 1938, *L. Constance 2309* A (GH), A, B (UC); 7.6 mi SE of Quincy (at Spring Garden Overpass) on road to Blairsden, 11 Jul 1969, *L. Constance 3875* (UC), (GH); Sierra Nevada, about 2 miles from Spring Garden on road to Quincy, 20 Sep 1974, *J.T. Howell 50896* A - D (CAS); Sierra Nevada. 2 mi. northwest of Spring Garden, 16 Jun 1975, *J.T. Howell 51131* A, B (CAS); 2.65 mi W of Round Valley Reservoir dam, 23 Jun 1981, *J.T. Howell 54150* (NY); Plumas Nat’l Forest 7.6 mi. SE of Quincy (at Spring Creek Overpass) on road to Blairsden, 200 yards up steep serpentine slope from train track, 2 Jul 2012, *D.P. Morin 36* A - F (NMC); Plumas Nat’l Forest. 16.6 road miles S on La Porte Rd to E turnoff toward Onion Valley Reservoir 30 yards from La Porte Rd as slope steepens, 2 Jul 2012, *D.P. Morin 39* A (NMC); 1/2 mi. S of La Porte Rd, 1/2 mi SE (and above) serpentine cliffs. 1 mi NW of Pilot Peak, 3 Jul 2012, *D.P. Morin 44* A, B, C, D (NMC), E, F (DUKE); Plumas Nat’l Forest. 0.5 air mi WSW of Onion Valley Reservior. +/- 16.75 road mi from La Porte turnoff N in East Quincy. Above cliffs ESE of La Porte Rd., 3 Jul 2012, *D.P. Morin 45* F, H, L (NMC); Plumas NF; along all roads near Clear Creek NE of Mine Pit, E of Clear Creek, 8 Jun 1983, *J.H. Robertson 17217* (UNR); At junctions of roads 26N18, 26N92 and 27N92, ca. 0.5 mi east of Long Valley Mine, ca. 4 air miles west of Greenville, 5 Jul 1981, *M.S. Taylor 3649* A, B (CAS); S side of Hwy 70 ca. 200 yards S of Spring Garden Overpass ca. 7.5 mi SE of Quincy (T24N, R10E, S25; type locality), 2 Jun 1981, *M.S. Taylor 3827* (MO); East side of 25N17, ca. 0.125 mi south of Bean Creek, ca. 0.25 mi north of jct. 25N17 with 25N81 (Old Mt House Rd.). Ca. 3.5 air mi NW of Meadow Valley, 22 Jun 1981, *M.S. Taylor 3944* (CAS); Both sides of spur road off 26N18, ca. 0.5 mi southeast of Long Valley Mine, ca. 4 air mis west of Greenville, 18 May 1982, *M.S. Taylor 4471* A, B (CAS); South side of 27N92, ca. 1.5 air mi southeast of Canyon Dam, 18 May 1982, *M.S. Taylor 4474* A, B (CAS); South side of 27N92, ca. 2 air mi southeast of Canyon Dam, 18 May 1982, *M.S. Taylor 4478* A, B (CAS); Both sides of road 27N92, near Goldstripe Mine, ca. 2.25 mi SE of Canyon Dam., 18 May 1982, *M.S. Taylor 4479* A, B, C (CAS); North side of 204, ca. 1 air mi southeast of Long Valley Mine, ca. 4 air mis west of Greenville, 1 Jun 1982, *M.S. Taylor 4582* C (CAS); County Rd 204 ca. 2 mi W from Round Valley (site); Crescent Mills 7.5 USGS quadrangle; NE .25 Section 8, 26 May 2004, *D.W. Taylor 19075* A (ORE), A, B (JEPS). Sierra County: About 3/4 mile (air) east of Lake Delahunty, about 2 miles (air) northeast of Gibsonville (39°45'01"N by 120°52'39.6"W), 28 Jun 2006, *L. Ahart 12874* (JEPS); West slope of the northern Sierra Nevada, 1.8 miles (linear) NE of Gibsonville on the road to Johnsville, 2.3 miles WNW of Mount Etna, 13.2 miles SSE of Quincy, Plumas National Forest, 26 Jun 2009, *P.J. Alexander 1046* A (BRY), B, D - F (NMC); Plumas Nat’l Forest. Open serpentine knoll 300 m WSW of Lake Delahunty. 50 m S of sign “Entering and Welcome to Delahunty Lake.” 1 mi E from La Porte Rd on McCrea/Johnsonville Rd. Large, healthy population, 3 Jul 2012, *D.P. Morin 40* A - D, H (NMC), E, F, G (DUKE), I, J (MO); Plumas Nat’l Forest. From La Porte Rd, 1.8 mi road mi E on McRea/Johnsonville Rd 2ft from N side of road in washed out open serpentine slope, 3 Jul 2012, *D.P. Morin 41* A, B, C, D (NMC); Plumas Nat’l Forest. From La Porte Rd, 1.8 road mi E on McRea/Johnsonville Rd 70 ft from S side of Rd., 3 Jul 2012, *D.P. Morin 42* A - D (NMC).

**Figure 6.**
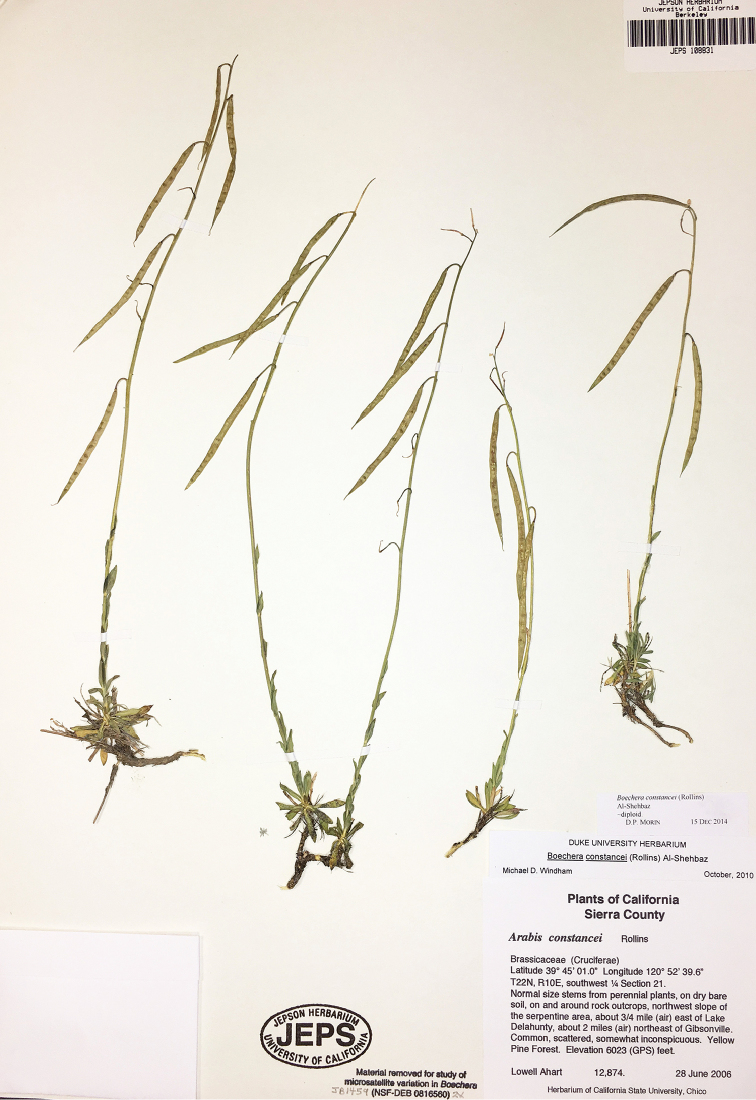
*Boechera constancei*, Ahart 12,874 (JEPS).

#### Boechera duriuscula

Taxon classificationPlantaeBrassicalesBrassicaceae

(Greene) D.P. Morin comb. nov.

urn:lsid:ipni.org:names:77178481-1

Figures 3 , 7

≡ Arabis duriuscula Greene, Pittonia 4: 191. 1900. **Type: U.S.A. California.** Nevada County: Donner Lake, *Michener s.n.* (1893) and *Sonne s.n.* (1882). The repository of the Sonne syntype(s) is unknown at this time; the Michener syntype deposited at NDG has been included in our morphological and molecular analyses and is here designated as lectotype.

##### Description.

Plants long-lived perennials, with ±woody caudices occasionally raised above ground level, lacking crowded, persistent leaf bases; herbage without an obvious bluish cast. Fertile stems 1–3(–4) per caudex branch, each arising from a basal rosette; lower parts pubescent to densely pubescent with short-stalked, 2–3(4) rayed trichomes 0.1–0.3 mm. Leaves at stem bases oblanceolate, 1.7–5.8 mm wide, entire, ciliate with 2–3(–4) rayed trichomes to 0.07–0.40 mm; cauline leaves (4–)6–12, occasionally concealing stem proximally, the uppermost glabrous, with auricles (0)0.3–1.4 mm. Inflorescences mostly unbranched, 6–12 flowered; mature fruiting pedicels 9–17mm, reflexed, distinctly geniculate proximally, but otherwise straight, glabrous. Flowers pendent at anthesis; sepals glabrous; petals pale lavender or whitish with rose tips apically 4.5–6.0 mm long × 2.0–2.5mm wide; anthers with mostly well formed, narrowly ellipsoid, symmetrically tricolpate pollen; ovules 20–30 per fruit. Fruits 3–7(–10) cm long × 2.0–2.5 mm wide, pendent, straight to somewhat curved, with minutely undulate edges; apical angle of fruit valve <25° (measured from base of style to 5 mm proximate); style glabrous, 0.2–1.2 mm. Seeds uniseriate, 2.5–5.5 × 1.8–3.5 mm; wing continuous, 0.8–1.5 mm wide.

##### Distribution, habitat and phenology.

*Boechera duriuscula* is distributed in the Sierra Nevada from Kaiser Crest in Fresno County north to Mt. Hough in central Plumas County California. A few populations have been documented in Washoe, County Nevada, in the vicinity of Lake Tahoe. It is found on rocky or gravelly felsic substrates, often in association with *Abies magnifica*, *Pinus jeffreyi* and open *Wyethia* meadows at elevations from 2200–2750 m; flowering May–July.

##### Comments.

*Boechera duriuscula* is distinguished from *B. suffrutescens* s.s. by being persistently pubescent basally. It differs from *B. botulifructa* by having fruits that taper more gradually apically (< 25° versus ≥30° as measured from the apex to a point 5 mm proximal to it).

##### Specimens examined. California.

Alpine County: Armstrong Pass, 9 miles south of South Tahoe, 12 Aug 1978, *G.L. Stebbins 78149* A, C, D (CAS). El Dorado County: Summit area Echo Peak, 27 Jul 2012, *G.L. Smith 2497* (JEPS); ENE of Kyburz below Cup Lake near head of Tamarack Creek ca. 1.17 km SSE of the summit (9235) of Ralston Peak. T11N, R17E, Sec. 9. Lat.: 38°49'22"N Long.: 120°05'57"W (WGS84 Datum), 18 Jun 2002, *M.D. Windham 2579* A (MO), A (NMC). Fresno County: Kaiser Crest, 27 Jul 1914, *F.J. Smiy 621* (GH). Nevada County: Donner Lake, 1893, *E. Michener s.n.* (NDG); Truckee, Sierra Nevada Mountains, Jun 1892, *C.F. Sonne 9* A, B, C (UC); Sierra Nevada. Just W of Truckee near Donner State Park., 30 Jun 1965, *G.H. True 2142* A, B (CAS). Placer County: NE from Highway 267, travel 3.4 mi on Martis Peak Road toward Martis Peak Lookout, 1 Jul 2012, *D.P. Morin 32* A - E (NMC), F, G (DUKE), H, I, J (MO); Tahoe Nat’l Forest 3.5 air mi N of Lake Tahoe. 2.7 air mi NE of Brockway Summit. 3.7 road mi on Martis Peak Road from northern turnoff from Highway 267, 1 Jul 2012, *D.P. Morin 34* A, B, C, (NMC); Tahoe Nat’l Forest. Turnout on N side of Road to Martis Peak Lookout. +/- 2.6 air mi from Brockway Summit., 1 Jul 2012, *D.P. Morin 35* A, B, C, (NMC), D, E, F (DUKE), G, H, I (MO); Martis Peak, western flank near headwaters Monte Carlo Creek (T17N, R17E, S34, NE), 15 Jul 2005, *D.W. Taylor 19408* (JEPS). Plumas County: North side of the summit of Mount Elwell, ±0.1 miles N of the highest point, ±1.8 miles SE of Mt. Washington, ±14 miles NE of Downieville, northern Sierra Nevada, Plumas National Forest, 19 Apr 2009, *P.J. Alexander 864* A, B (NMC); Sierra Nevada. Jamison Creek., 27 Jun 1951, *J.T. Howell 27628* A, B, C (CAS); Summit ridge of Mt. Hough, 11 Jul 1967, *J.T. Howell 43348* A (GH), (CAS); Mount Hough Summit, ca. 7 mi NNE of Quincy., 5 Aug 1982, *M.S. Taylor 4927* A, B, C (CAS). Sierra County: At Verdi Peak Lookout, Verdi Range., 14 Jul 1970, *J.T. Howell 5522* A, C, D (CAS); Sierra Nevada. Near Mount Etna, 4 mi E of Gibsonville. Sierra-Plumas county line., 20 Jul 1975, *A. Tiehm* B (CAS). Tuolumne County: 2 mi w Sonora Pass, 27 Jul 2005, *R.C. Rollins 2993* (UC), (RSA). **Nevada.** Washoe County: Peavine Mountain, S of Murpheys meadow, T18N R20E sec. 17, 8 Aug 1974, *A. Tiehm 505* (UNR); Sierra Nevadas, Carson Range, N side of Galena Creek on the south side of Mt. Rose, 5 Aug 1983, *A. Tiehm 8279* B, C (CAS).

**Figure 7.**
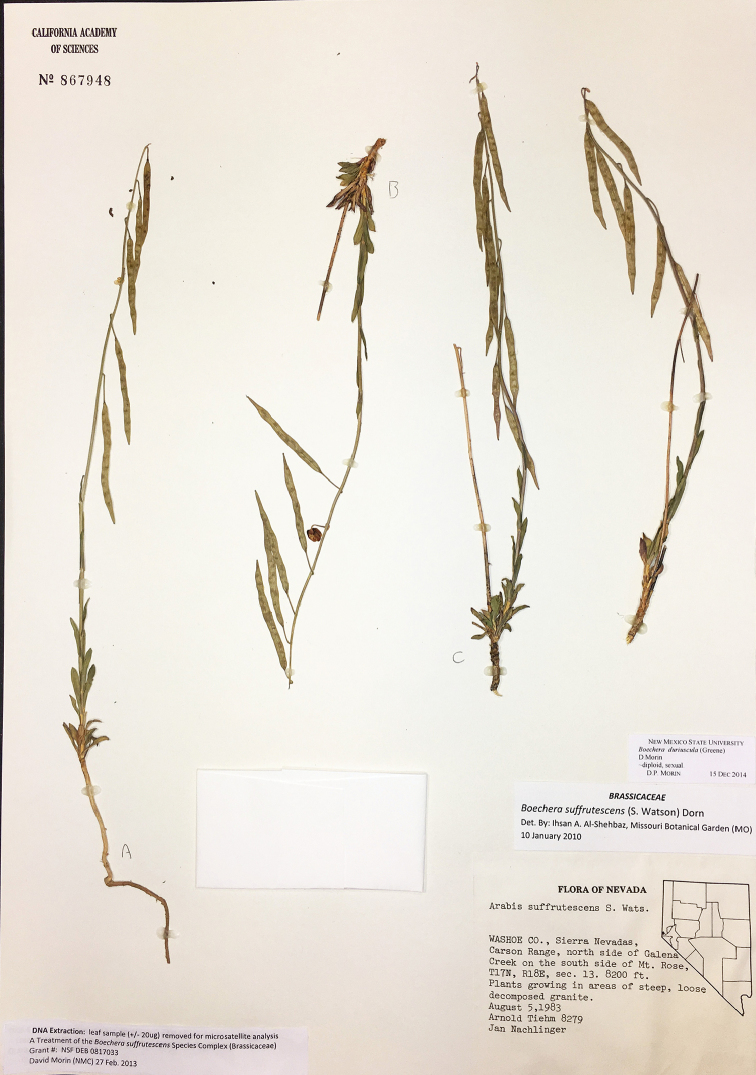
*Boechera duriuscula*, Tiehm and Nachlinger 8279 (CAS).

#### Boechera rollei

Taxon classificationPlantaeBrassicalesBrassicaceae

(Rollins) Al-Shehbaz, Novon 13: 389. 2003

Figures 3 , 8

≡ Arabis rollei Rollins, Harvard Pap. Bot. 4: 43. 1993. **Type: U.S.A. California.** Siskiyou County: Divide between the Applegate and Klamath rivers, Red Butte-Kangaroo Mt, Lilypad Lake –Towhead region, 4 Aug 1983, *W.E. Rolle 831* (holotype: GH!; isotypes: JEPS!, MO). GH holotype image – http://kiki.huh.harvard.edu/databases/specimen_search.php?mode=details&id=67660

##### Description.

Plants long-lived perennials, with woody caudices raised above ground level 1–5 cm, lacking crowded, persistent leaf bases; herbage without an obvious bluish cast. Fertile stems usually 1 per caudex branch, arising from centre of basal rosettes, glabrous throughout. Leaves at stem bases oblanceolate, 3–8 mm wide, entire, ciliate proximally with 1–3 rayed trichomes 0.2–0.7 mm, blade surfaces glabrous or sparsely pubescent with short-stalked, 2–4 rayed trichomes 0.2–0.4 mm; cauline leaves 6–12, occasionally concealing stem proximally, the uppermost glabrous, with auricles 0.5–2.5 mm. Inflorescences unbranched, 3–7 flowered; fruiting pedicels 4–8 mm, arched, curved proximally (not geniculate), glabrous. Flowers divaricate-ascending at anthesis; sepals glabrous; petals creamy-white and occasionally blushed lavender, 8–11 mm long × 2.0–2.5 mm wide, glabrous; anthers with mostly well formed, narrowly ellipsoid, symmetrically tricolpate pollen; ovules 14–22 per fruit. Fruits 35–65 mm × 2.0–3.5 mm, pendent to reflexed, not appressed to rachises, often secund, straight to somewhat curved, with undulate edges, glabrous; apical angle of fruit valve <25° (measured from base of style to 5 mm proximate); style persistent 0.6–1.2 mm. Seeds uniseriate, 3–4 × 1.5–2.0 mm; wing distal and proximal, 0.3–0.6 mm wide.

##### Distribution, habit and phenology.

Populations of *B. rollei* are known from the Klamath Mountains Province primarily in the vicinity of Lilypad Lake in Siskiyou County, California. However, a single individual from Beaver Creek in Jackson County, Oregon has been confirmed morphologically and genetically as *B. rollei*. It is noteworthy that the collector of this specimen (F.W. Hoffman 2551) mentioned that it came from serpentine soil, to which *B. rollei* appears to be restricted. The species is often associated with *Pinus jeffreyi* and *Calocedrus decurrens* on sparsely forested slopes at elevations of 1600–1800 m; flowering June–Aug.

##### Comments.

*Boechera rollei* is distinguished from the three S2X clusters of *B. suffrutescens* s.l. by its showier flowers with longer (8.0–11.0 vs. 4.5–6.0 mm) petals and narrower (0.3–0.6 vs. 0.8–1.5 mm) seed wings. It is easily distinguished from *B. constancei* by its markedly auriculate cauline leaves, shorter (0.6–1.2 vs. 1.5–5.5 mm) styles and lack of herbage with bluish cast.

##### Specimens examined.

California. Siskiyou County: Below the Pacific Crest Trail on the W side of Lilypad Lake, ±0.5 mile S of Red Butte, ±5.5 miles N of Seiad Valley, Siskiyou Mountains, Rogue River National Forest, 24 Jul 2008, *P.J. Alexander 869* A, D, E (DUKE), C, D, E (NMC); On the Pacific Crest Trail ±0.7 miles SSW of Cook and Green Pass, ±1.9 miles E of Red Butte, ±7 miles NNE of Seiad Valley, Siskiyou Mountains, Rogue River National Forest, 17 Apr 2009, *P.J. Alexander 873* (MO), A, B, C (NMC); 0.1 mi SW of border between Siskiyou National Forest and Klamath National Forest. NE facing slope above Lilypad Lake on Pacific Crest Trail. 0.7 air miles SSW of Red Butte summit. 1/4 mi SW of Lilypad Lake, 29 Jun 2012, *D.P. Morin 13* A, B (NMC) (these represent the DNA vouchers for samples A – O); Divide between the Applegate and Klamath Rivers, Red Butte-Kangaroo Mt.-Lilypad Lake-Towhead Lake region (T47N, R12W, S13), 8 Apr 1983, *W.E. Rolle 831* A - D (GH), A (JEPS); Red Buttes Wilderness Area about 3/5 mi S of Towhead Lake, or ½ mi W of Lily Pad Lake. T47N R12W Sec. 13 NW1/4 of SW1/4., 8 Apr 2009, *W.E. Rolle 1538* (DUKE); Near Lilypad Lake, Red Butte and Kangaroo Mt., 27 Aug 1985, *W.E. Rolle s.n.* (TEX), (GH), A, B (JEPS), A, B (CAS). **Oregon.** Jackson County: Upper Beaver Creek, 10 Jul 1948, *Hoffman 2551* (UC).

**Figure 8.**
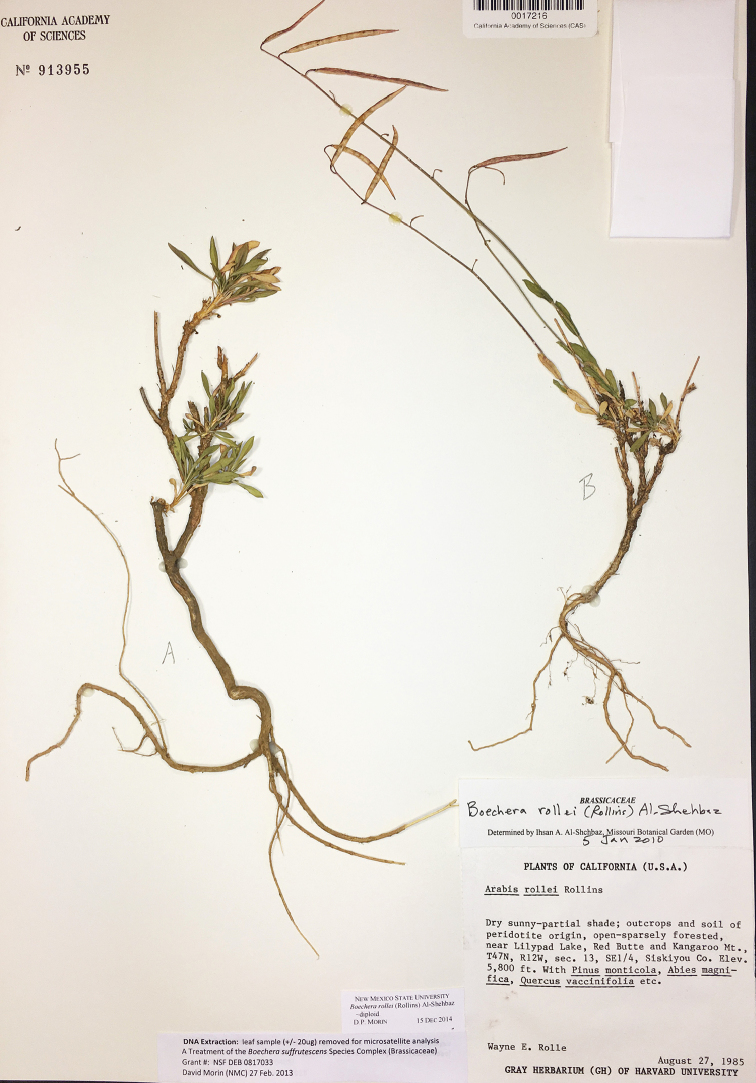
*Boechera rollei*, Rolle s.n. (CAS).

#### Boechera suffrutescens

Taxon classificationPlantaeBrassicalesBrassicaceae

(S. Watson) Dorn, Brittonia 55: 3. 2003

Figures 3 , 9

##### Type.

**U.S.A. Oregon.** Wallowa County/Baker County: Bluffs of Snake River and vicinity, 1881, *W.C. Cusick 919* (holotype: GH!; isotype: ORE).

GH holotype image – http://kiki.huh.harvard.edu/databases/specimen_search.php?mode=details&id=27057

##### Description.

Plants long-lived perennials, with woody caudices raised above ground level 1–5 cm, lacking crowded, persistent leaf bases; herbage without an obvious bluish cast. Fertile stems usually 1 per caudex branch, arising centrally from basal rosettes, lower parts glabrous or glabrate with 1–2(3) rayed trichomes (0.1–0.3 mm). Leaves at stem bases narrowly oblanceolate to obsubulate, 1.5–6.0 mm wide, entire, not ciliate or rarely with simple trichomes to 0.7 mm, blade surfaces usually glabrous or sparsely pubescent with 1–3(4) rayed trichomes, but occasionally plant herbage basally pubescent with short-stalked 1–4(5)-rayed trichomes (0.07–0.40 mm) if stressed or sterile; cauline leaves (4–)6–12, sometimes concealing stem proximally, the uppermost glabrous, with auricles 0.8–4.5 mm long. Inflorescences mostly unbranched, 6–12-flowered; fruiting pedicels 4–18 mm, reflexed, usually distinctly geniculate proximally but otherwise straight, glabrous. Flowers pendent at anthesis; sepals glabrous; petals purple or whitish with rose tips, 4.5–6.0 mm long × 2.0–2.5 mm wide, glabrous; anthers with mostly well formed, narrowly ellipsoid, symmetrically tricolpate pollen; ovules 20–30 per fruit. Fruits 1.7–5.5 cm long × 3.3–4.0 mm wide, reflexed, pendent, occasionally appressed to rachises, often secund, straight to somewhat curved, with undulate edges, glabrous; apical angle of fruit valve 15°–23° (measured from base of style to 5 mm proximate); style persistent, 0.4–1.2 mm in length. Seeds uniseriate, 2.5–5.5 × 1.8–3.5 mm; wing continuous, 0.8–1.5 mm wide.

##### Distribution, habit and phenology.

*Boechera suffrutescens* is distributed north and east of the Great Basin; concentrated in the vicinity of the Snake River Gorge (Hells Canyon), but extending from Grant County, Oregon to central Idaho on steep, rocky, basaltic substrates in alpine and subalpine ecozones at elevations from 1800–2500 m; flowering from May–July.

##### Comments.

Although geographically isolated from the other S2X species of the complex, *B. suffrutescens* s.s. is the least distinct morphologically. The most useful character for distinguishing this species is that individuals usually have basal leaves that are glabrate, with a few 1(–2) rayed trichomes scantily dispersed along the margins and apices. However, plants are occasionally encountered that are pubescent basally with 1–3(–4) rayed trichomes. These individuals often appear stressed or lack flowering stems, suggesting that pubescence may be more prevalent amongst plants growing in unfavourable environments. On the holotype specimen, one of each morphotype is present and the plant lacking a flowering stem is pubescent. All other taxa in the complex are consistently pubescent basally. On robust individuals of *B. suffrutescens* s.s., the basal leaves are generally narrower and the fruits are generally wider than those of the other S2X taxa.

##### Specimens examined.

**Idaho.** Adams County: Confluence of Wildhorse River and No Business Cr. On N & W exposures, 13 May 1987, *D. Atwood 12561* (GH). Valley County: In basaltic outcrop on E side of high ridge W of Cascade. Payette NF, 15 Jul 1937, *R.C. Rollins 13852* (UC). Wash County: Seven Devils Mts., 10 Jul 1899, *M.E. Jones 6164* A, B (RSA). Washington County: Middle slopes of Hitt Mountain, 15 Jun 1943, *C.H. Christ 14044* (OSC); Dry hillside above Spring Creek. Ida Range 5 W Twsp. 14 North, 22 Jun 1940, *R.J Davis 2184* A, B, C (GH). **Oregon.** Grant County: West rim of High Lake Basin, Blue Mts., 4 Aug 1946, *B. Maguire 26497* (UC). Union (Baker/Wallowa) County: Bluffs of Snake River and vicinity, 1881, *W.C. Cusick 919* (GH); Stony hills near Snake River, 26 May 1898, *W.C. Cusick 1808* (UC); Overlooking Hells Canyon from west. Northeast of Hells Canyon Overlook. E +/- 100 yards from NFD 490. Take unpaved NFD 490 NE +/-3mi from Wollawa Mountain Loop, 22 Jun 2012, *D.P. Morin 10* A, B, C, D (NMC), E, F (DUKE); ESE facing slope 1/4 mi NE from dirt Hat Point Road on E side of Saddle Creek Campsite. Overlooks the Seven Devils Mtns and Saddle Creek to the east, a tributary to Snake River, 25 Jun 2012, *D.P. Morin 11* A (NMC); Hat Point Road and Saddle Creek Campground. Overlooks the Seven Devils Mtns and Saddle Creek Canyon to the E., 26 Jun 2012, *D.P. Morin 12* A, B, C (NMC); ENE facing rocky basalt on W side of Wallowa Mountain Loop. +/- 1.5 mi N of turnoff to Lick Creek Campground. 16.5 air miles SE of Joseph., 25 Jun 2012, *D.P. Morin 14* A, B, C, D (NMC), E, F, G (DUKE); Snake River Canyon near the mouth of Battle Cr., 12 Jul 1933, *M.E. Peck 17616* (OSC); SE of Enterprise near crest overlooking McGraw Creek E of Forest Route 490 ca. 0.25 road mi NE of the parking area at Hells Canyon Overlook, 22 Jun 2012, *M.D. Windham 4110* (DUKE); SE of Enterprise on slope above Lick Creek along Wallowa Mountain Loop Rd. ca. 14.1 road mi W of its junction with Upper Imnaha Rd., 22 Jun 2012, *M.D. Windham 4132* (DUKE).

**Figure 9.**
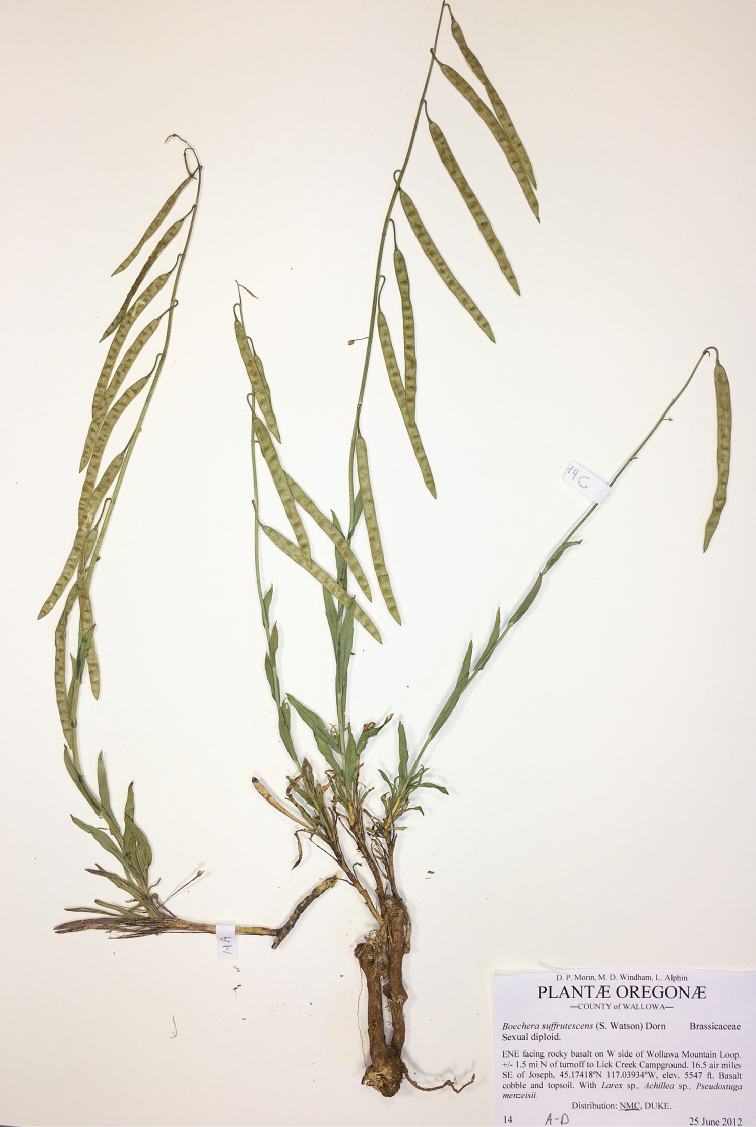
*Boechera suffrutescens*, Morin, Windham, Allphin 14 (NMC).

## Supplementary Material

XML Treatment for Boechera botulifructa

XML Treatment for Boechera constancei

XML Treatment for Boechera duriuscula

XML Treatment for Boechera rollei

XML Treatment for Boechera suffrutescens

## Appendix 1

Additional specimens examined for individual samples that were deemed singeltons (2X), apomictic diploid (A2X), triploid (A3X) or polyploid (≥ 4x). All individuals were collected in the U.S.A. If present, suffixes (A, B, C etc.) indicate specific individuals from a collection event in which vouchers of leaf samples from multiple individuals were collected. All populations are represented by voucher specimens deposited in the herbaria indicated (acronyms from *Index Herbariorum*).

**Boechera cf. constancei (A2X): California.** Plumas County: Plumas Nat’l Forest. SSE aspect, 1 mi NW of Pilot Peak. 0.5 mi WNW of Onion Valley Resevoir. 0.4 mi WSW from W turnoff 23N24 from La Porte Rd., 2 Jul 2012, *D.P. Morin 38* A - D (NMC), E, F (DUKE); La Porte Highway. +/-17 mi road mi S of East Quincy, 3 Jul 2012, *D.P. Morin 43* A, B (NMC); Plumas Nat’l Forest. 0.5 air mi WSW of Onion Valley Reservior. +/- 16.75 road mi from La Porte turnoff N in East Quincy, 3 Jul 2012, *D.P. Morin 45* A - E, G, I (NMC), J, K (DUKE). Sierra County: West slope of the northern Sierra Nevada, 1.8 miles (linear) NE of Gibsonville on the road to Johnsville, 2.3 miles WNW of Mount Etna, 13.2 miles SSE of Quincy, Plumas National Forest, 9 Apr 2009, *P.J. Alexander 1046* (NMC). **Boechera cf. suffrutescens (A2X)** California. Lassen County: 3.9 car mi NE of Blue Lake. 6.5 air mi WNW of Hat Mtn., 30 Jun 2012, *D.P. Morin 26* A, B (NMC). Modoc County: NE slope Emerson Peak; Warner Mountains, 23 Jul 1946, *A.M. Alexander 5054* A, B (UC), B (GH); Cedar Peak, Warner Mts., 1 Jul 1940, *M. Ownbey 2147* (CAS), (WS); Warner Mountains, E face of Emerson Peak, Emerson Creek drainage Emerson Peak; Warner Mountains, Emerson Peak, 5 Aug 1989, *D.W. Taylor 10436* A (JEPS). Plumas County: Summit ridge of Thompson Peak, Diamond Range; Canadian Zone forest, areas of volcanic rock, 31 Jul 1973, *J.T. Howell 50117* B (CAS). Shasta County: Vicinity of Magee Peak (Summit of Crater Peak, Lassen National Forest, Thousand Lakes Wilderness); Lassen National Forest, Thousand Lakes Wilderness, 24 Aug 1975, *D.W. Taylor 5430* A, B (JEPS). Siskiyou County: Ridge W of Baldy Mt. L. O., 6 mi by air W of Happy Camp, Siskiyou Mts., 2 Jul 1952, *P.A. Munz 17911* A, B (RSA). Trinity County: North slope of North Yolla Bolly Peak, Yolla Bolly Mtns. Line of Tehama and Trinity Counties., 18 Jul 1951, *P.A. Munz 16636* A, B, C (RSA). **Nevada.** Humboldt County: Humbolt NF, Santa Rosa Range, ca. 50 air mi. NNW of Winnemucca, a. 5 mi W of Paradise Valley. Mostly on the ridge between Singas Creek and Morey Creek drinages. Access was gained by driving up the dirt road to Singa. A few plants found on the granitic, 26 Jul 2009, *C.D. Bailey 306* (DUKE). Washoe County: Fox Mountain approx. 26.5 air mi NNW of Gerlach, NV. Approximately 0.33 mi N (downslope) from Fox Mountain Summit (radio tower access route Old Camp Canyon Rd.) descending into NE facing gully, E facing aspect., 30 Jun 2012, *D.P. Morin 28* A, - E, G (NMC); Sierra Nevadas, Carson Range, N side of Galena Creek on the south side of Mt. Rose, 5 Aug 1983, *A. Tiehm 8279* A (CAS), A, B, C (GH); Granite Range, Fox Mt. on the NW end of the range, just N of the peak, 30 Jul 1986, *A. Tiehm 10818* A, C (OSC), A, B, C (RSA), A, B (GH). **Oregon.** Deschutes County: Paulina Lake., 30 Jun 1931, *J.T. Howell 7097* A, B (GH). Grant County: High Lake Basin, Malheur NF, Blue Mts, 4 Aug 1946, *B. Maguire 26497* (TEX); Malheur NF, along trail near road 1640, near bend in road on S side of Strawberry Mt., 19 Aug 2010, *N. Otting* 16442 A (OSC); About 5 mi SW of Anthony Lake, on granitic soil, 17 Jul 1952, *C.L. Hitchcock 19715* A, B (RSA). Harney County: SE of Frenchglen near crest of Steens Mountain along North Loop Road ca. 1.3 road mi WSW of the turnoff to Kiger Gorge Overlook. Lat.: 42.69940N; Long.: 118.59760W (WGS84 Datum), 4 Aug 2009, *M.D. Windham 3829* (DUKE); Steens Mtn. along road on W side of McCoy Creek, 12 Jul 1979, *Wright 1127* (OSC). Klamath County: SE side of Crater Lake on slope overlooking Kerr Valley along Rim Drive ca. 0.9 road mis ESE of its junction with Pinnacles Road. Lat.: 42.90910N; Long.: 122.05950W (WGS84 Datum), 8 May 2009, *M.D. Windham 3843* (DUKE). Lake County: Fremont N.F.; E of McDowell Peak (T37S, R22E, S20, NE, NW); in rocky areas, 24 Jul 1991, *B. Rittenhouse 733* (OSC). **Washington.** Yakima County: Mt. Adams, 15 Aug 1882, *J.T. Howell, s.n.* (OSC). Unassigned ***Singletons* (2X): California.** Plumas County: 1 mi NW of Pilot Peak, 14 mi NE of La Porte, 8 July 1986, *Ahart 5356* (CAS); Jameson Creek W of Johnsville; rocky outcrop in yellow pine forest, 28 May 1985, *D. Anderson 2805* (GH). Shasta County: Trinity Mountains; Grey Rocks; ca. 10 air mis WSW of Dunsmuir (T38N, R5W, S21, SW), 9 Jul 1993, *D.W. Taylor 13824* (JEPS). Siskiyou County: Along Forest Service road 17, 0.5 mi north of summit at county line. China Mountain Quad., 17 Jul 1980, *T.W. Nelson 6094* (CAS). **California.** Siskiyou County: Red Butte-Kangaroo Mt.-Lilypad Lake- Towhead Lake region, T47N R12W, Sec. 13, 8 Apr 2004, *W.E. Rolle 831* (MO). **Oregon.** Baker County: Largest rocks of Pine Creek near Snake River (possible topotype), May n/a, *W.C. Cusick* (ORE). **Washington.** Unknown County: Falcon Valley, 18 May 1884, *W.N. Suksdorf s.n.* A, B, C (UC). **Boechera cf. suffrutescens Complex (A3X): California.** El Dorado County: Tallac Trail between Lake Gilmore and Camp Lake Tahoe Region, 24 Jul 1907, *R.L. Pendleton 1126* (UC). Elmore County: 10 mi W of Featherville Divide above Trinity Lakes, 25 Jul 1944, *C.L. Hitchcock 10347* A, B (UC). Plumas County: About 1/2 mi S of Bucks Lake (N of a poor logging road), 29 Jun 1994, *L. Ahart 7409* (JEPS). **California.** Plumas County: Sierra Nevada. On road from Round Valley to Long Valley, 31 May 1974, *W. Dakan* A, B (CAS); Summit ridge of Thompson Peak, Diamond Range, 31 Jul 1973, *J.T. Howell 50117* (CAS); East side of 25N17, ca. 0.125 mi south of Bean Creek, ca. 0.25 mi north of jct. 25N17 with 25N81 (Old Mt House Rd.). Ca. 3.5 air mi NW of Meadow Valley., 22 Jun 1981, *M.S. Taylor 3944* A (CAS); Red Hill Lookout, ca. 3 air mi NE of Belden., 25 Aug 1981, *M.S. Taylor 4237* (CAS); North side of 27N92, ca. 2 air mi SE of Canyon Dam, 18 May 1982, *M.S. Taylor 4476* (CAS); Between spur road and 27N92 NW of Goldstripe Mine ca. 4 air mi W of Greenville (T27N, R8E, S36, SW), 1 Jun 1982, *M.S. Taylor 4579* (MO). Shasta County: Thousand Lake Basin; forest floor of the interlake region, 11 Jul 1932, *F.W. Peirson 10151* (RSA); s of Burney; Thousand-Lake Basin, 11 Jul 1932, *F.W. Peirson 10151* A, B, C (UC). Sierra County: West slope of the northern Sierra Nevada, 1.8 miles (linear) NE of Gibsonville on the road to Johnsville, 2.3 miles WNW of Mount Etna, 13.2 miles SSE of Quincy, Plumas National Forest., 9 Apr 2009, *P.J. Alexander 1046* (NMC). Siskiyou County: South Fork of Salmon River near Big Flat., 21 Jul 1937, *J.T. Howell 13204* A, B (GH); Caribou Basin, Salmon-Trinity Alps, 24 Jul 1937, *J.T. Howell 13379* (GH); East side of Hiway 93 between Calahan and Cecilleville at Carter Summit Trailhead. +/- 11 mi SW of Calahan, 28 Jun 2012, *D.P. Morin 20* A, B, D - J (NMC), C (DUKE); N of Hancock Lake (W side of Red Hill, vicinity of English Peak); Salmon Mountains, Marble Mountain Wilderness Area, English P, 16 Aug 1968, *F.W. Oettinger 540* (UC); Small ridge near Wolverine Lake, Marble Mts., 25 Sep 1985, *W.E. Rolle s.n.* (GH); Trinity Alps, above S Fork of Salmon R, along Yellow Rose Mine trail, top of Pass between Yellow Rose and Dorleska mines, 15 Jul 2005, *R.C. Wenk 206* A, B (CAS); Mt. Eddy, 12 Jun 1976, *J. Whipple 981* A (GH); Caribou Lake Salmon/Trinity Alps Primitive Area, 27 Jul 1955, *I.L. Wiggins 13534* A, B (CAS). **Idaho.** Adams County: Micah Summit, 9 mi S of Cabin Creek Campground, W side of dirt road on embankment. 4.75 mi due W of Cascade Resevoir. Near intersection of NFD 206 and NFD 207. 3/4 mi ENE of Indian Point Lookout, 21 Jun 2012, *D.P. Morin 8* B, C, D (NMC), E, F (DUKE); South of Micah Summit, on W side of Anderson Creek Rd. (NFD 206); 0.2 mi W of Weiser River, 21 Jun 2012, *D.P. Morin 9* A, B, C (NMC), D, E, F (DUKE); SE of Council near Mica Saddle along FR 206 ca. 100 meters NNE of its junction with FR 207, 21 Jun 2012, *M.D. Windham 4104* (DUKE); SE of Council below Mica Saddle along Forest Route 206 ca. 1.2 road miles S of its junction with Forest Route 207, 21 Jun 2012, *M.D. Windham* (DUKE). Blaine County: West side of Galena Pass on the N side of ID Hwy 75, N end of the Smoky Mountains, ±23 miles NW of Ketchum, Sawtooth National Forest, 20 Apr 2009, *P.J. Alexander 884* A - E (NMC); Dry gravelly hillside of Galena Summit, 29 Jul 1941, *A. Cronquist 3521* A (GH); 7.3 mi N of Ketchum, 19 Jun 1979, *R.J. Davis 79276* B (GH); Above Galena, Central Idaho, Jul 1895, *L.T. Henderson 3537* A, B, C (ORE); Iron Mt., 5 mi W of Martin, 26 Jun 1938, *C.L. Hitchcock 3826* A, B (GH); 7.3 mi N of Ketchum, 19 Jun 1979, *R.C. Rollins 79276* A, B (UNR), A (UC); Rocky ridge ca. 1.5 mi. N of Galena Summit, between Stanley and Kethcum, 27 Jun 1986, *R.C. Rollins 86133* (TEX); N side of Galena Summit, off State Hwy. 75, between Stanley and Ketchum, 27 Jun 1986, *R.C. Rollins 86117* (TEX), C (GH); Ca. 1.5 mi N of Galena Summit, off State Hwy. 75, between Stanley and Ketchum, 27 Jun 1986, *R.C. Rollins 86133* A - E (GH). Camas County: Crouch Summit, 7 mi N of Soldier, 5 Jul 1965, *C.L. Hitchcock 23813* A, B (CAS). Custer County: Ridge above Mill Creek, 12 mi W of Challis, 8 Jul 1941, *A. Cronquist 2966* A, B (GH); Ca. 0.5 mi NE of Toxaway Lake. 10 mi WSW of Obsidian; Sawtooth Mts., 11 Aug 1944, *C.L. Hitchcock 5739* A (UC), B (GH). Elmore County: On highest slope of Trinity Peak; 17 mi. N of Fall Creek, 12 Jul 1950, *J.H. Christ 20051* A - F (OSC); Sawtooth Prmitive Area; headwaters of Middle Fork Boise River above Atlanta. Rocky slope ca. 4 mi S of Spangle Lakes, 19 Jul 1944, *C.L. Hitchcock 10167* A, B (UC). Idaho County: Hibbs Cow Camp, Seven Devils Mts., 26 Jun 1940, *M. Ownbey 2083* A (UC), A, B (OSC), A, B (GH). Ketchum County: Road towards Galena Summit, 29 mi N of Ketchum, 19 Jun 1979, *R.C. Rollins 79279* A, B, E (GH). Owyhee County: Above Sawpit Creek. Ca. 3 mi SW of Silver City, 12 Jul 1951, *W.H. Baker 7891* (GH). Valley County: Box Lake ca. 9 air mi NE of McCall, E-facing bare granite slope on opposite side of ridge SE of lake; overlooking Lick Creek Road, 27 Jul 1989, *B. Ertter 8793* A, B (UC); Elk Summit, Range 8E, Township 21N. Moist sliding soil, 7 Jul 1940, *R.J. Davis 2652* A, B, C (UC); In basaltic outcrop on E side of high ridge W of Cascade, Payette NF, 15 Jul 1937, *J.W. Thompson 13852* A, B, C (GH). Washington County: Rush Creek, 7 Jul 1899, *M. Jones 6164* (UC); Rush Creek, 5 Aug 1899, *M.E. Jones s.n.* A, B (RSA). **Nevada.** Washoe County: Granite Range, Fox Mt. on the NW end of the range, just N of the peak, 30 Jul 1986, *A. Tiehm 10818* B (OSC). **Oregon.** Baker County: Ekhorn Range, Hunt Mtn.; Pine Creek drainage, 20 Jul 1986, *E. Joyal 1235* (OSC); Rocky granitic hillside along Wood River. 10 mi N of Ketchum, 29 Jul 1941, *A. Cronquist 3481* A, B (GH). Grant County: About 5 mi SW of Anthony Lake, 17 Jul 1952, *C.L. Hitchcock 19715* A, B (RSA). Harney County: Steens Mts. Opposite Devine Ranch, 5 Jul 1896, *J.B. Leiberg 2514* A (GH). Wallowa County: Between Douglas Lake and Moccasin Lake, 23 Aug 1946, *B. Maguire 27157* (UC). **Nevada.** Washoe County: Granite Range, Fox Mountain on the NW end of the range, just N of the peak, 30 Jul 1986, *A. Tiehm 10818* A (CAS). **Oregon.** Josephine County: Near the summit of Lake Mountain, Oregon Caves National Monument & vic., 3 Jul 1949, *Baker 265* (WTU); Sand Ridge, Lake Mountain Trail; Oregon Caves National Monument & vicinity, 16 Aug 1949, *Baker 646* (UC).

## Supplementary materials

Supplementary material 1 Representative pollen morphologies Data type: measurement Explanation note: **A**. S2X pollen of *B. rollei* (Morin 13, NMC). **B**. Potentially functional pollen from an A3X individual (Morin 8, NMC). **C**. Representative malformed non-functional pollen from A2X and some A3X individuals (Christ 20051, OSC). This is an open access article distributed under the terms of the Creative Commons Attribution License (CC BY 4.0), which permits unrestricted use, distribution, and reproduction in any medium, provided the original author and source are credited.Morin DP, Alexander PJ, Beck JB, Windham MD, Bailey CD

Supplementary material 2 Analysis 1 Data type: statistical data Explanation note: **A**. STRUCTURE bar plots with the highest likelihood for K = 5, 6, and 8 respectively. Instability was consistently noted across iterations at all values of K. B. An MDS plot demonstrates distinctness in TL-ro in three dimensions and TL-su in two dimensions. Calculations for optimal K were ambiguous and inconsistent, as were clustering patterns with regard to the **B**. constancei clusters (TL-co, PLSI-co and CD-co), particularly with regard to TL-co. MDS plots do not represent analyses, but are a visual interpretation of the dataset. TL-su and TL-ro are the most distinct and coherent clusters based on allele sharing distance. This is an open access article distributed under the terms of the Creative Commons Attribution License (CC BY 4.0), which permits unrestricted use, distribution, and reproduction in any medium, provided the original author and source are credited.Morin DP, Alexander PJ, Beck JB, Windham MD, Bailey CD
