# Supplementary figures and images for: Deciphering the sexual diploid members of the Boechera suffrutescens complex (Brassicaceae, Boechereae)
Source: PhytoKeys. 2018 May 2;(98):15–50. doi: 10.3897/phytokeys.98.24296 (PMC5943444; doi:10.3897/phytokeys.98.24296)

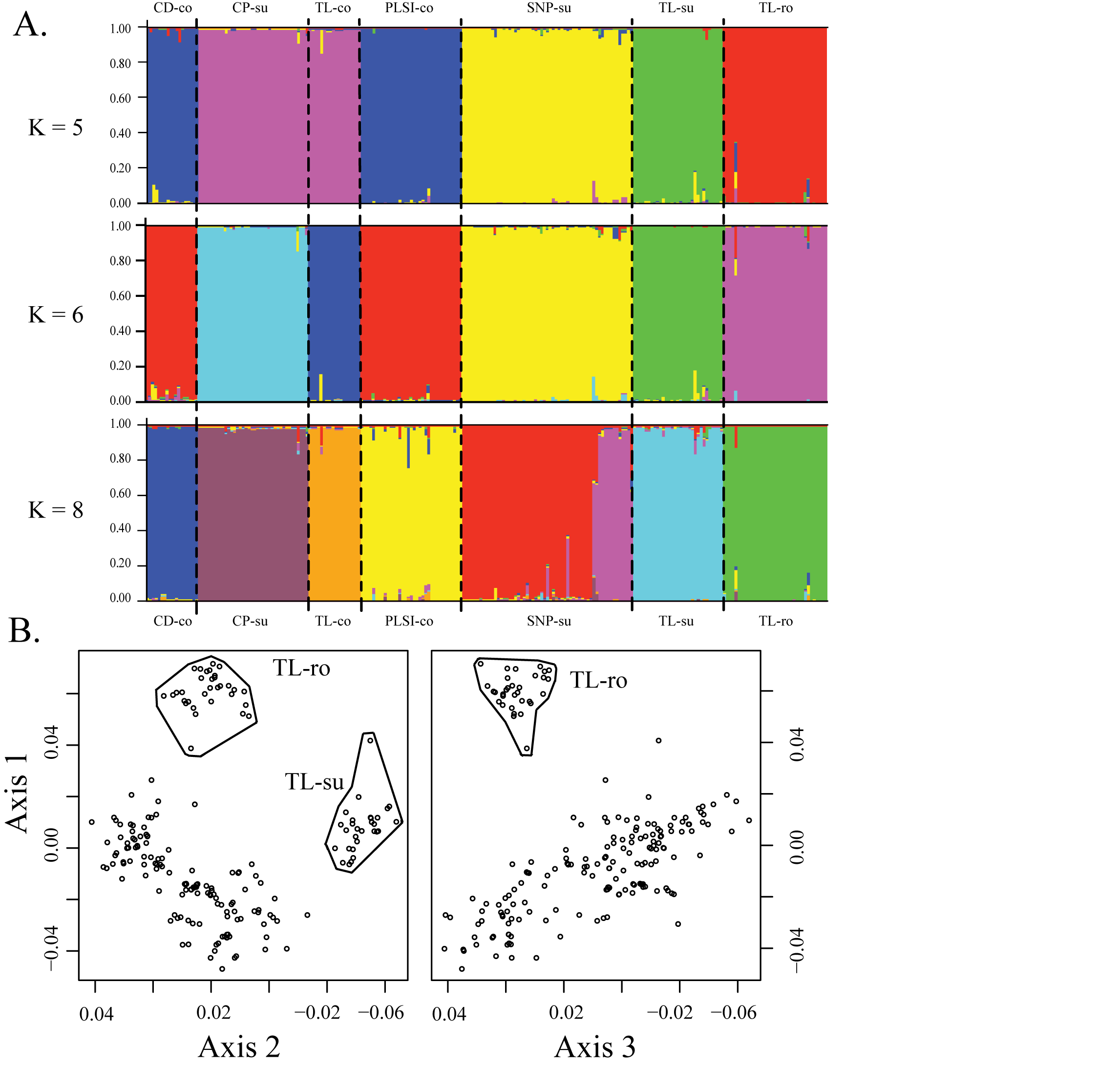

Supplement: Supplementary material 1 — Representative pollen morphologies [file phytokeys-98-015-s002.tif]
